# Breeding and biotechnology approaches to enhance the nutritional quality of rapeseed byproducts for sustainable alternative protein sources- a critical review

**DOI:** 10.3389/fpls.2024.1468675

**Published:** 2024-11-11

**Authors:** Anandhavalli Manikandan, Saraladevi Muthusamy, Eu Sheng Wang, Emelie Ivarson, Sudha Manickam, Rajeswari Sivakami, Manikanda Boopathi Narayanan, Li-Hua Zhu, Ravikesavan Rajasekaran, Selvaraju Kanagarajan

**Affiliations:** ^1^ Department of Genetics and Plant Breeding, Centre for Plant Breeding and Genetics, Tamil Nadu Agricultural University, Coimbatore, Tamil Nadu, India; ^2^ Department of Plant Breeding, Swedish University of Agricultural Sciences, Lomma, Sweden; ^3^ Department of Plant Biotechnology, Centre for Plant Molecular Biology and Biotechnology, Tamil Nadu Agricultural University, Coimbatore, Tamil Nadu, India; ^4^ Centre for Plant Breeding and Genetics, Tamil Nadu Agricultural University, Coimbatore, Tamil Nadu, India

**Keywords:** rapeseed byproducts, nutritional enhancement, anti-nutritional factors, microbial fermentation, seed storage proteins, breeding strategies, genome editing

## Abstract

Global protein consumption is increasing exponentially, which requires efficient identification of potential, healthy, and simple protein sources to fulfil the demands. The existing sources of animal proteins are high in fat and low in fiber composition, which might cause serious health risks when consumed regularly. Moreover, protein production from animal sources can negatively affect the environment, as it often requires more energy and natural resources and contributes to greenhouse gas emissions. Thus, finding alternative plant-based protein sources becomes indispensable. Rapeseed is an important oilseed crop and the world’s third leading oil source. Rapeseed byproducts, such as seed cakes or meals, are considered the best alternative protein source after soybean owing to their promising protein profile (30%–60% crude protein) to supplement dietary requirements. After oil extraction, these rapeseed byproducts can be utilized as food for human consumption and animal feed. However, anti-nutritional factors (ANFs) like glucosinolates, phytic acid, tannins, and sinapines make them unsuitable for direct consumption. Techniques like microbial fermentation, advanced breeding, and genome editing can improve protein quality, reduce ANFs in rapeseed byproducts, and facilitate their usage in the food and feed industry. This review summarizes these approaches and offers the best bio-nutrition breakthroughs to develop nutrient-rich rapeseed byproducts as plant-based protein sources.

## 1 Introduction

United Nations predicts that the global population will exceed 9.5 billion by 2050. Agri-food Innovation Council (2019) estimates that global protein consumption will double within the next 40 years, increasing from 473 Metric tons (Mt) to 944 Mt by 2054 (Agri-Food Innovation Council, 2019; available at https://nrc.canada.ca/sites/default/files/2019-10/Plant_protein_industry_market_analysis_summary.pdf). This substantial increase in protein demand necessitates an annual increment of 14% in protein production. Hence, identifying novel alternative protein sources is crucial to supplement the growing protein needs. In addition to animal proteins, potential alternative plant protein sources must be exploited to meet the population’s growing needs. Traditional animal protein sources such as eggs, poultry, fish, and dairy provide complete protein diets containing all the essential amino acids. However, its high fat and low fiber composition poses significant health risks such as obesity, gastro-esophageal reflux disease (GERD), cardiovascular diseases, type 2 diabetes, sleep disorders, and non-alcoholic fatty liver disease (Neuenschwander et al., 2023). Moreover, the production of animal-based proteins is environmentally unsustainable, consuming high levels of energy and natural resources and increasing the emission of greenhouse gases. These factors have increased the demand for vegan-friendly and non-animal protein sources in food, beverages, and snacks. Also, consumers are interested in healthy dietary choices with high protein and low fat content, which has driven the market for plant-based proteins to gain momentum in recent years.

Rapeseed (*Brassica napus* L.) emerges as a promising alternative plant-based protein source, ranking second after soybean with promising nutrient profiles (Bell, 1993; Feng and Zuo, 2007; Nega and Woldes, 2018). It is the third leading source of vegetable oil globally, followed by soybean and palm oil; rapeseed meal (RSM) is the second most produced protein after soybean meal. Rapeseed has versatile uses, including oil for human consumption, protein-rich seed cakes or meals for animal feed, biodiesel extraction, and various industrial applications. The oil of commercial rapeseed varieties comprises 6%–14% α-linolenic acid, 50%–66% oleic acid, and less than 7% saturated fatty acids and is considered a healthy choice for human consumption (Ghazani and Marangoni, 2013). The byproducts of rapeseed oil extraction, such as cake and meal, are potential protein sources for human and animal consumption, with crude protein content ranging from 30%–60%. Compared to cereals, RSM contains higher concentrations of essential amino acids such as tryptophan, threonine, lysine, and sulfur-containing methionine and cysteine, making it the best alternative source for demanding protein needs (Le Thanh et al., 2019). However, anti-nutritional factors (ANFs) in the seed cake or meal limit its implementation in human diets and animal feeds.

## 2 Nutrient profile of rapeseed byproduct

The nutrient profile of rapeseed byproducts is comparable to soybean meal. RSM’s crude protein content indicates its immense potential as a substitute for the plant-based protein diet for food and feed (**Table 1**).

**Table 1:** Comparison of the feed quality of rapeseed cake, rapeseed meal, and soybean meal.

Component (in %)	Rapeseed cake^a^	Rapeseed meal^a^	Rapeseed protein isolate^c^	Dehulled soybean meal^b^
Crude protein	35.7	38.6	94.39	48.1
Crude fiber	11.4	11.8	–	3.4
Ash	7.2	7.3	1.36	6.00^a^
Neutral detergent fibre	33.3	20.7	–	7.1
Acid detergent fibre	26.0	16.8	–	5.0

Adapted from ^a^(Feng and Zuo, 2007), ^b^(Bell, 1993), ^c^(Jia et al., 2021).

The postprandial biological value of rapeseed protein isolate (RPI) is 84%, which is higher than milk (82%) and soy protein (80%) (Bos et al., 2007). The balanced amino acid profile (**Table 2**) contributes to its biological value. Legumes such as green gram, peas and lentils are the primary plant-based protein sources for the human diet. Legumes are richer in lysine than cereals. However, they are lower in lysine and sulfur-rich amino acids than animal proteins (Yang et al., 2023). Nevertheless, unlike soybean meal, RSMs provide sulfur-rich amino acids (**Table 2**). However, high temperatures in the protein extraction methods might invoke the Maillard reaction and influence the level of available lysine content in rapeseed cakes or meals (Newkirk et al., 2003; Almeida et al., 2014; Salazar-Villanea et al., 2016).

**Table 2:** Comparison of amino acid profile between rapeseed and soybean meal.

Amino acid	Amino acid requirements for adults^a^	Rapeseed meal*	Soybean meal
Essential amino acid profile			
Arginine	–	21.5	32.7
Histidine	1.5	9.1	11.1
Isoleucine	3.0	12.6	18.1
Leucine	5.9	25.4	34.5
Lysine	4.5	20.9	29.0
Methionine	1.6	6.9	5.8
Phenylalanine	3.0	14.4	22.5
Threonine	2.3	15.2	17.3
Tryptophan	0.6	5.2	6.9
Valine	3.9	16.3	18.8
Lysine availability	–	1.0	0.9
Lysine: Cp. g/100g	–	5.5	6.3
Non- essential amino acid profile			
Alanine	–	16.4	19.7
Aspartic acid	–	25.1	50.4
Cysteine	–	8.1	6.1
Glutamic acid	–	62.0	83.4
Glycine	–	18.7	19.8
Proline	–	22.1	23.9
Serine	–	14.7	23.1
Tyrosine	–	10.6	17.4
Total amino acids	–	331.8	443.0

Adapted from (Le Thanh et al., 2019). Analysed nutrient content (g/kg) of soybean meal and five rapeseed meal samples.

a Adapted from (WHO/FAO/UNU, 2007). The requirement of amino acid expressed as g/100g protein.

*Average value of 5 different rapeseed meals from 5 commercial rapeseed crushers in AB, SK, and MB, Canada.

The digestibility of the protein determines the extent of protein utilization in human food. Rapeseed proteins showed poor *in vitro* digestibility (83%) compared to casein (97%) (Savoie et al., 1988). The real ileal digestibility (RID) of RPI is the lowest (84%) when compared to milk (95%) and soybean (91.5%) (Bos et al., 2007). The high fiber content in the rapeseed cake or meal is a concern for their use as feed in animal husbandry as it is four times higher than the soybean meal (**Table 1**) (Bell, 1993; Feng and Zuo, 2007), affecting meal digestibility. Additionally, the gross energy of RSMs and metabolizable energy (ME) is lower than that of soybean meal (Blair and Reichert, 1984; Bell, 1993). ME of RSM in swine and poultry is reported to be 9.33 MJ/Kg and 7.41 MJ/Kg, respectively (Feng and Zuo, 2007). However, the ME of soybean meal was reported to be ~15-18 MJ/Kg (Lopez et al., 2020). The higher lignin content reduces the feed digestibility in RSMs, which is a prominent limitation of its application.

## 3 Rapeseed byproducts as a novel protein source in food and feed

Seed storage proteins (SSPs) are unique proteins found in the seeds of rapeseed and accumulated during seed maturation. Almost 90% of rapeseed proteins are storage proteins, made up of 11S cruciferin of the cupin superfamily and 2S napin of the prolamin superfamily (Wanasundara, 2011). About 32%-53% of the proteins in the rapeseed seeds are made up of cruciferin, a globulin-type protein (Malabat et al., 2003). Cruciferin is characterized by its large hexameric structure, with each subunit connected by a disulfide-bridged α and β subunits (Wanasundara et al., 2017). Napins are albumin proteins and account for 25%-45% of the total proteins in the rapeseed seeds (Malabat et al., 2003). The primary structure of a napin protein comprises 111 to 180 amino acids, while the secondary structure comprises four helices. Two subunits, small 4.5 kDa and large 10 kDa, are linked by two disulfide bonds (Wanasundara, 2011).

The properties of these proteins have immense potential for the food industry. The solubility of proteins is a functional requirement for a food-based protein system (Wanasundara et al., 2017). The solubility of napin is more than 90% in the pH range of 2-10, whereas cruciferin exhibits lower solubility in this pH range in relation to napin (Wanasundara et al., 2017). Cruciferin has better emulsifying properties than napin, whereas napin has better foaming capability than cruciferin. The emulsifying ability facilitates the use of rapeseed proteins in the production of mayonnaise and salad dressings. The poor gelling ability of the proteins limits their use in the food industry by reducing the firmness of the products (Thompson et al., 1982). However, enzymatic modification of canola protein isolate (CPI) with transglutaminase can improve the gelling ability (Pinterits and Arntfield, 2008). Food-grade canola protein products are commercially available under the names Puratein^®^ (precipitated micelle protein of near neutral pH protein extracted with salt, >90% protein, cruciferin protein mainly), Supertein™ (protein remained soluble after micelle formation; Burcon Nutrascience, >90% protein, napin mainly). The Protein Digestibility Corrected Amino Acid Score (PDCASS) values of Supertein™ and Puratein^®^ (Burcon Nutrascience protein products) were 0.61 (61%) and 0.64 (64%), respectively (Wanasundara et al., 2017). Utilizing rapeseed protein products in foods could result in a balanced protein diet (**Table 3**).

**Table 3:** Utilizing rapeseed/canola as a source of protein in the food industry.

Food products	Formulations	Extraction process	Comments	Reference
Bread	RPI and RPC	RPI and RPC obtained from rapeseed flour by successive water, HCl, and NaOH extraction process	5% RPI and RPC with 0.5% Atmul 124 increased loaf volume by 10 to 15% and 12%, respectively. Crust color of RPI or RPC containing bread was slightly darker than that of the control. Supplementing RPI or RPC levels higher than 6% resulted in darker crust.	(Kodagoda et al., 1973)
Rice flour bread	3%, 6%, and 9% CPC	Alkaline extract	6% CPC in rice flour successfully mimicked the property of gluten and resulted in gluten free bread with satisfactory functional and sensory characters. Substituting higher percentage of CPC resulted in browning of bread.	(Salah et al., 2019)
Gluten free biscuits	3%, 6%, and 9% CPC or CPI	Electro-activation of extracted canola protein	Improved the thickness and diameter of the biscuits thereby reducing the spread ratio. Reduced hardness and improved aeration, springiness and crunchiness of biscuits.	(Gerzhova et al., 2016)
Sausage	2% and 4.5% RPC	Complex extraction process	Sausages with 2% RPC resulted in satisfactory functional properties and sensory characters. Whereas sausages with 4.5% of RPC resulted in browning, oily and strawy off-flavors due to high amounts of secondary plant metabolites.	(Von Der Haar et al., 2014)
	RPI	Acidic pre-extractions and alkaline protein extractions	RPI inclusion reduced the cooking loss and the resulted sausages had comparable functional and sensory qualities.	(Von Der Haar et al., 2014)
	RPC	Alcohol washed aqueous extracts	Steamed RPC improved the taste of sausages and had a characteristic aroma but the texture and color of sausage was poor.	(Yoshie-Stark et al., 2006)
Meat patties	3% RPC	RPC extracted from rapeseed flour with 2% hexameta-phosphate	RC reduced shrinkage % of beef patties. RC inclusion adversely affected the color and flavor of the patties.	(Thompson et al., 1982)
Mayonnaise	7% and 14% CPH	Alkaline extract of CM hydrolyzed using protease	7% and 14% CPH can substitute egg yolk at 20% and 50% respectively. CPH increased particle size and reduced viscosity. Inclusion of CPH caused reddish-brown color of mayonnaise.	(Aluko and McIntosh, 2005)

RPI, Rapeseed Protein Isolate; RPC, Rapeseed Protein Concentrate; CPI, Canola Protein Isolate; CPC, Canola Protein Concentrate; CPH, Canola Protein Hydrolysates; CM, Canola Meal.

Despite their potential in the food industry, the ANFs significantly impact the sensory characteristics of rapeseed proteins, including flavor and color. Moreover, the extraction procedures highly affect the protein’s functional and sensory characteristics, which limits its usage in food formulations (Von Der Haar et al., 2014). Additionally, the presence of allergens such as napins and 2S albumins, which can cause IgE-mediated allergic responses (Puumalainen et al., 2006; Moreno and Clemente, 2008), limits their usage in food products (Monsalve et al., 2001; Murtagh et al., 2003; Puumalainen et al., 2006). Because of their stable disulfide bridges, napins and 2S albumins can resist proteolysis, high temperatures, low pH, and stay intact (Murtagh et al., 2003; Jyothi et al., 2007). Due to their resistance to degradation, they can remain in the gut for prolonged periods and may stimulate an immunological response, leading to allergic reactions (Pantoja-Uceda et al., 2004; Puumalainen et al., 2006). This resistance demands techniques to modify the protein structures to improve digestibility, which might decrease its allergic reactions and increase its potential use in the food industry. However, few rapeseed protein isolates (RPIs) have been considered safe for consumption by the Food and Drug Administration (FDA) (So and Duncan, 2021), and further research is needed to enhance their applications.

Rapeseed byproducts offer a cheap source of protein supplements for animal diets (**Table 4**). However, the presence of ANFs produces off-flavors and odors, which reduce the palatability of these byproducts and adversely affect the animal’s growth and development.

**Table 4:** Rapeseed/Canola byproducts as a source of protein for animal feed.

S.no	Livestock/Poultry/Fishes/ Shrimps	Type of byproduct	Composition	Beneficial effects	Negative effects	Reference
1	Cattle	CM	2.5 kg/head/day	Cattles fed with CM and grain-based pellet feeds showed comparable carcass characters. The meat quality and palatability were unaffected by the inclusion of CM.	The diet reduced the Hot Standard Carcass Weight (HSCW) by 2.5%.	(Lynch et al., 2024)
2	Cattle	Solvent extracted CM	17.1% DM	MUN (Milk Urea Nitrogen) value was the lowest in CM indicating, efficient protein utilization. CM increased the DMI (Dry Matter Intake), FA (Fatty Acids) and reduced the enteric gas emissions. The milk yield and the plasma AA (amino acids) profiles were comparable to SBM.	–	(Lage et al., 2021)
3	Cattle	Ground canola seed	14% DM	Increased the long chain fatty acids. MUN value lesser than control indicating efficient protein usage.	Milk fat and protein was lesser than the control.	(Chichlowski et al., 2005)
4	Cattle	Double-low rapeseed meal (DLRSM)	15% DLRSM diet and 15% DLRSM + 32 g/d rumen protected lysine (DLRSML)	DLRSML increased milk yield, 4% Fat Corrected Milk (FCM), Energy corrected Milk (ECM), and protein yield compared with the SBM. Inclusion of DLRSM had the same effect as the SBM. DLRSML might be better than DLRSM.	DLRSM inclusion could possibly affect the palatability of dairy cows. Requirement of additional lysine indicates that lysine is a limiting amino acid in the meal.	(Liu et al., 2016)
5	Cattle	RSM	RSM and 1:1 ratio of RSM and faba bean at low and high inclusion rates	Inclusion of RSM increased the milk yield, milk protein yield, milk fat yield, concentration of EAA (Essential Amino Acids) in plasma and showed effective protein digestion.	Inclusion of faba bean reduced the effective protein utilization and reduced the milk protein.	(Puhakka et al., 2016)
6	Weaned Pigs	Expeller pressed canola meal (EPCM)	0, 50, 100, 150 or 200 g/kg of EPCM replacing SBM	Substituting SBM with 200 g EPCM showed similar Net Energy (NE) and SID (Standardized Ileal Digestible) amino acid content without reducing growth performance.	The increased fibre content in EPCM decreased digestibility.	(Landero et al., 2012)
7	Growing finishing pigs	Expeller pressed RSM	18% and 16% inclusion of expeller pressed rapeseed and faba bean (RSM/FB)	The growth rate, feed intake and carcass traits remained unaffected when RSM/FB was substituted for SBM. The substitution resulted in high levels of glycine and glutamine. Feeding RSM/FB improved pork color and increased the concentration of free amino acids, and sweet tasting metabolites.	Reduced the Polyunsaturated Fatty Acids (PUFA) content of the meat.	(Grabež et al., 2020)
8	Gestation and lactation stage sows	Solvent extracted CM	300 g/kg	Showed satisfactory reproductive performance. CM inclusion increased the abundance of gut lactic acid bacteria in sows.	Reduction in sow body weight.	(Velayudhan et al., 2018)
9	Broiler chicken	CM	0%, 10%, 20%, 30%, and 40% of CM replacing SBM	Dietary RSM up to 16.7% can be utilized in feed without compromising growth performance. Crude Protein (CP) digestibility was unaffected with diets including 20% of CM. Maximum villus height was observed in diets including 23.6% of CM, indicating better nutrient absorption.	Increasing the inclusion levels lower dry matter’s digestibility and hinder growth and performance.	(Gopinger et al., 2014)
10	Broiler chicken	RSM	RSM was added at two DCP (Dry Crude Protein) levels: 15.8% and 17.2%, replacing SBM	–	Adversely affected the growth performance and gut morphology.	(Qaisrani et al., 2020)
11	Turkey	Low-glucosinolate RSM	0, 60, 120, and 180 g/kg of RSM	Low glucosinolate RSM increased FCR and PUFA.	Drip loss and Warner-Bratzler shear force values decreased in turkeys fed with 180 g/kg of RSM. RSM impaired triiodothyronine secretion which affected the quality of meat.	(Mikulski et al., 2012)
12	Geese	RSM	0%, 4%, 8%, 12%, and 16% of RSM replacing soya bean meal	Dietary RSM up to 16% can be utilized in feed without compromising growth performance.	–	(Fu et al., 2021)
13	Rose snipper	Solvent extracted CM	150-450 g/kg of CM protein	Growth, hematological parameters, and body composition were unaffected by including low proportion of CM.	CM inclusion beyond 300 g/kg affected growth of the fish. The ANFs in the meal affected Apparent Digestibility Coefficients (ADC) of dry matter and energy.	(Hernández et al., 2020)
14	Grass carp	CM	CM (340 g/kg) and CM with supplements (CMS) (340 g/kg+ lysine+ methionine)	The hepatosomatic index, relative gut length, intestosomatic index and intestinal folds height were significantly improved in fish fed with CMS diets.	CM without lysine and methionine supplements showed reduced the digestibility.	(Jiang et al., 2016)
15	Rainbow trout	Canola protein isolate (CPI)	25%, 50%, 75% and 100% of CPI replacing FM	Diets with 75% CPI increased growth response, feed intake and feed efficiencies.	–	(Slawski et al., 2013)
16	Farmed Tilapia Strain of *Nile Tilapia*	CM	0%, 15%, 30%, 45%, 60%, and 75% of CM replacing FM	Diets with 75% of CM can be utilized without compromising the growth performance.	–	(Luo et al., 2012)
17	Japanese seabass	Roasted CM	10%-50% of roasted CM replacing FM	Diets with <20% roasted CM can be utilized as feed without compromising the growth performance.	Increasing the levels of CM in feed reduced growth, survival rate and serum lysozyme. The ANFs in the meal altered the activity of the digestive enzymes and affected the digestibility.	(Cheng et al., 2010)
18	Catfish	CM	0%, 12%, 24%, 36%, and 48% of CM replacing SBM	Diets with 36% of CM increased weight and 48% of CM increased FCR.	48% CM reduced the weight, Protein Efficiency Ratio (PER), and Protein Retention Value (PVR).	(Webster et al., 1997)
19	Shrimp	High fibre canola meal (CMHF) and Low fibre canola meal (CMLF)	Replacing menhaden meal protein with CMHF and CMLF at 150, 300 or 450 g/kg of dietary protein	Diets with CMHF up to 150 g/kg did not adversely affect the growth and development.	Higher inclusions reduced the potassium levels, survival, growth and development of the shrimp.	(Lim et al., 1997)
20	Kuruma shrimps	CM	4:6 blend of CM and SBM replacing the fish meal at approximately 70%, 85% and 100%	Diets up to 85% of CM and SBM (4:6) did not affect the growth performance, feed utilization, protein gain, protein retention, protease activity of juvenile kuruma shrimp. Including CM and SBM blend in feed can reduce the need for FM from 40% to only 6%.	Supplementing methionine, lysine and phytase enzyme is necessary to maintain the diet quality.	(Bulbul et al., 2016)

CM, Canola Meal; RSM, Rapeseed Meal; SBM, Soybean Meal; FM, Fish Meal; DM, Dry Matter.

## 4 ANFs in rapeseed byproducts

**Figure 1:**
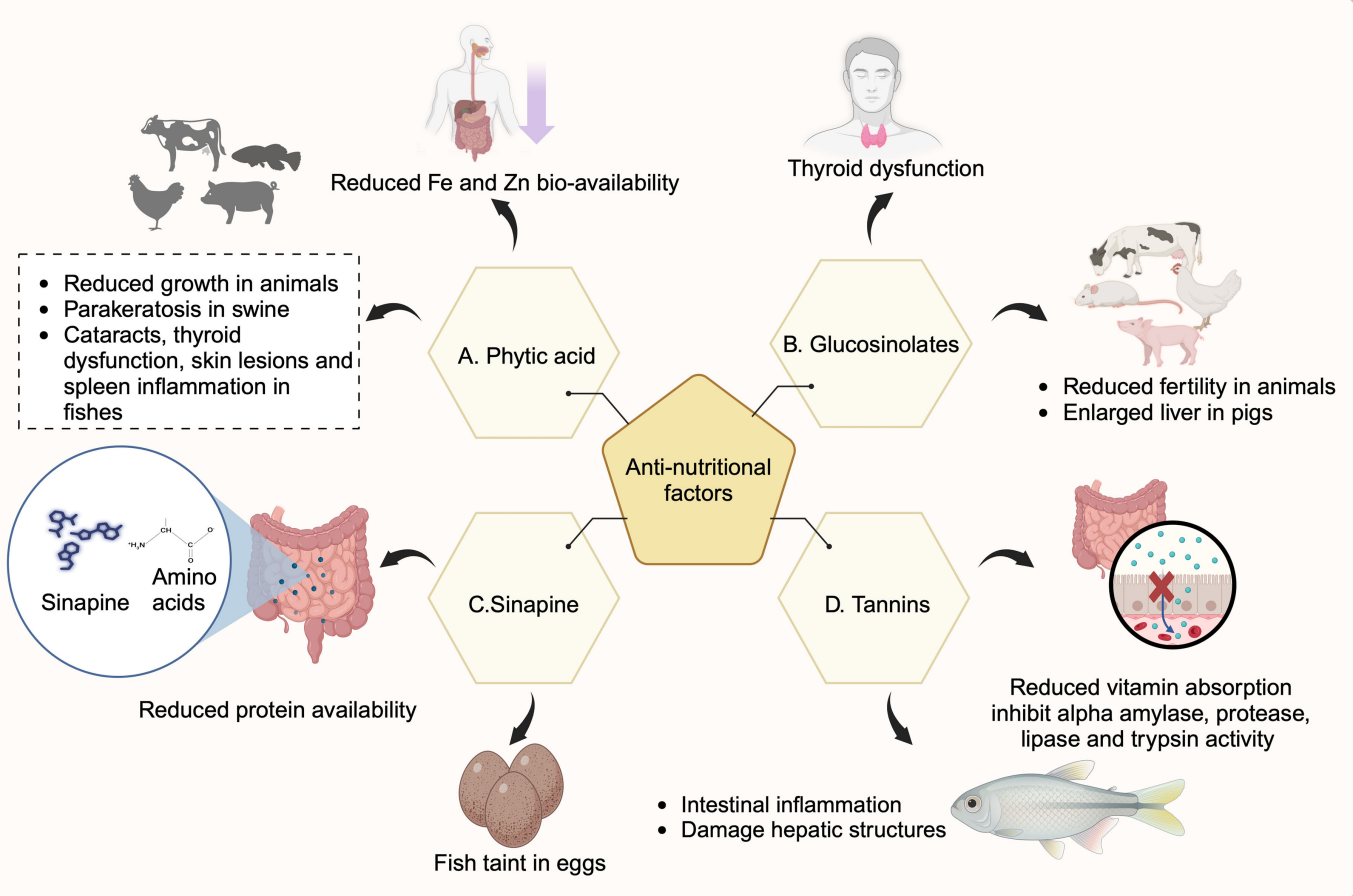
Effects of anti-nutritional factors (ANFs) in humans and animals. Rapeseed meal contains ANFs such as phytic acid, glucosinolates, tannins, and sinapines. **(A)** Phytic acid binds to metal ions such as iron and zinc and reduces their bioavailability. They cause thyroid dysfunction, cataracts, skin lesions, spleen inflammation in fish, and parakeratosis in swine. **(B)** Sinapines bind to amino acids and hinder their gut absorption, thereby reducing bioavailability. They also cause fish taints in eggs. **(C)** Glucosinolates cause thyroid dysfunction in humans, reduce fertility in animals and cause enlarged liver in pigs. **(D)** Tannins inhibit alpha-amylase, protease, lipase, and trypsin activities and affect protein digestion. Tannins cause intestinal inflammation, damage hepatic structures in fish, and impair vitamin absorption in rats (Created with BioRender.com).

ANFs are secondary metabolites and play an important role in plant defense against biotic and abiotic stresses. Any external environmental stress triggers an increase in secondary metabolite levels as a defense mechanism to cope with stress. However, they are the major problem in implementing rapeseed byproducts for food and feed consumption (**Table 5**). The ANFs at higher concentrations bind with the nutrients and reduce their bioavailability (Makkar, 2003) (**Figure 1**), causing adverse effects on the animal’s growth and development (Jansman, 1993; Mawson et al., 1994; Makkar, 2003). The major ANFs in rapeseed byproducts include glucosinolates, phytic acid, tannins, and sinapines.

**Table 5:** Major anti-nutritional factors (ANFs) in rapeseed meal.

ANFs	Compounds	OSR meal*	NTCM^c^	TCM^c^
Glucosinolates (μmol/g)	3-butenyl	0.88^b^	3.40	1.94
	4-pentenyl	0.11^b^	0.67	0.38
	2-hydroxy-3-butenyl	2.015^b^	6.28	3.64
	2-hydroxy-4-pentenyl	0.08^b^	0.20	0.20
	3-indolylmethyl	0.16^b^	0.58	0.22
	4-hydroxy-3-indoylmethyl	0.775^b^	4.2	0.78
Tannins (%)		1.5-3.0^a^	–	–
Sinapines (%)		0.6-1.8^a^	–	–
Phytic acid (%)		3.0-6.0^a^	–	–

Adapted from ^a^(Bell, 1993), ^b^(Le Thanh et al., 2019), ^c^(Newkirk et al., 2003).

NTCM, Non-Toasted Canola Meal; TCM, Toasted Canola Meal.

*Average of 5 rapeseed meal.

### 4.1 Glucosinolates

Glucosinolates (GSLs) are sulfur and nitrogen-containing compounds derived from glucose and amino acids as precursors, consisting of thioglucose and a sulfonated oxime attached to the chain-elongated amino acid (Blazevic et al., 2020). GSLs are classified as aliphatic, aromatic, or indolic based on the amino acid moiety type of side chain group (Halkier and Du, 1997). These are commonly found in *Brassica* family members, including rapeseed, cabbage, and mustard. The enzyme myrosinase hydrolyzes GSLs, yielding isothiocyanates and epi-thionitriles (Wittstock et al., 2003).

GSLs are responsible for the pungency of these plants, which reduces their consumer preferences and palatability as fodder. GSLs can alter thyroid hormone levels in humans and animals (Tripathi and Mishra, 2007; Felker et al., 2016). However, hydrolyzed GSL products are beneficial to humans. For example, sulforaphane and indole-3-carbinol exhibit anti-cancerous properties (Keck and Finley, 2004), while glucoraphasatin and glucoerucin are known to have anti-oxidant and anti-carcinogenic properties (Singh et al., 2020; Cedrowski et al., 2021; Sundaram et al., 2022). Also, glucoberteroin prevents non-alcoholic fatty liver disease (Kim et al., 2021). Conversely, the hydrolyzed products are highly toxic to animals, and RSM consumption reduces their fertility. These effects result from thyroid dysfunction in the mother, reduced iodine transfer to the fetus, transfer of goitrogenic compounds to the fetus, or a combined action of all these factors (Mawson et al., 1994). Furthermore, goitrogenic effects of GSLs, including reduced feed intake and enlarged liver, have been reported in pigs (Velayudhan et al., 2017; Lee and Woyengo, 2018; Lee et al., 2020).

### 4.2 Phytic acid (phytate)

Phytic acid (myo-inositol hexakisphosphate) was first identified in 1855 (Oatway et al., 2007). In mature seeds, phosphorous is stored mainly in the form of phytic acid (phytate) (Erdman, 1979), which acts as the primary phosphorus reserve in cereals and legumes and plays a vital role in germination and growth (Feizollahi et al., 2021). During germination, the phytase enzyme degrades phytates to release phosphorous, thus supplementing the growth and development (Pramitha et al., 2021). Phytate levels increase at the ripening stage and are usually located in the aleurone layer and pericarp of cereal grains, as well as in the endosperm of maize (O’Dell et al., 1972; Bohn et al., 2007). In oilseeds, phytate is distributed throughout the seed and inside the protein body membrane (Erdman, 1979).

Humans and monogastric animals lack the phytase enzyme, which is necessary to digest phytates. About 70% of phosphorous ingested by monogastric animals is excreted (Jorquera et al., 2008). Insoluble phytate–metal ion complexes are formed at neutral and basic pH, with the concentration of metals exceeding that of phytate (Maenz, 2001). Moreover, phytic acid is a potent chelating agent that binds to monovalent and divalent cations, reducing their bioavailability (Erdman, 1979; Schlemmer et al., 2009; Moran and Bedford, 2024). This chelation substantially limits the bioavailability of essential minerals such as iron and zinc in humans, leading to nutrient deficiencies (Kim et al., 1993; Reddy et al., 1996; Kim et al., 2007; Hunt and Beiseigel, 2009). For example, a diet high in phytic acid might increase the risk of anemia and decrease zinc absorption, especially in women. The elimination of phytic acid increased zinc bioaccessibility by 18.19% in low-protein soymilk and increased the bioaccessibility of calcium and iron by 31.20% and 30.03%, respectively, in high-protein soymilk (Lv et al., 2024).

Phytates inhibit the action of α-amylase and affect starch digestion (Knuckles and Betschart, 2006). Phytins in the aleurone layer of grains, when exposed to low gastric pH dissociate as phytic acid and free Ca^2+^ ions. The free Ca^2+^ ions bind with pepsin and compromise proteolysis (Moran and Bedford, 2024). By reducing the activity of digestive enzymes, phytate makes proteins resistant to proteolytic digestion, affecting amino acid absorption (Knuckles et al., 1985; Liu et al., 2009). In animal nutrition, dietary phytic acid reduces growth and development in broilers and pigs (Woyengo and Nyachoti, 2013). Broilers, when fed with casein and 1g of phytic acid, increased the excretion of Ca (187%), Mg (39%), Mn (87%), and Na (174%) (Cowieson et al., 2006). Also, phytic acid reduced the ileal amino acid digestibility in broiler chickens (Ravindran et al., 2006). In pigs, the addition of phytic acid to their diet has been associated with symptoms of parakeratosis and growth depression (Oberleas et al., 1962). Similarly, supplementing 2% phytic acid in feed reduced the body weight gain and feed intake and the growth of pigs up to 37% (Woyengo et al., 2012; Woyengo and Nyachoti, 2013). In Chinook salmon, diets that included phytic acid have been observed to affect thyroid, kidney and alimentary tract morphology. Also, cataracts and hypertrophy of pyloric caeca were increased (Kaiser et al., 2022). Similarly, grass carp fed with phytate lowered antimicrobial activity, caused head, kidney, and spleen inflammation, and increased skin lesions (Zhong et al., 2020). Thus, the presence of phytic acid in feed negatively impacts the growth and development of animals. However, adding phytase to the diet can hydrolyze the phytic acid and improve the bioavailability of nutrients.

### 4.3 Tannins

Rapeseed hulls mainly contain condensable tannins (Shahidi and Naczk, 1992). They can be categorized as hydrolyzable and condensed tannins. Tannins are known for their anti-viral, anti-bacterial, anti-oxidant, anti-parasitic, and anti-diarrheic properties (Tong et al., 2021). Their natural occurrence helps to control bloating in ruminants (Mangan, 1988). However, tannins are known to impart a bitter taste in feed and reduce its palatability. Also, tannins inhibit the activity of digestive enzymes such as trypsin, protease, lipase, and α-amylase (Horigome et al., 1988; Maitra and Ray, 2003; Xiao et al., 2015), resulting in reduced protein digestibility. The inclusion of 1.25% tannins in aquafeeds resulted in impaired digestion and protein metabolism (Yao et al., 2019). Similarly, including 0.6% tannic acid in the feed diets of Xiangdong black goats, significantly reduced the crude protein digestibility (Wang et al., 2022). Also, including 0.75% tannins in feed induced oxidative stress and injured hepatic antioxidant ability, resulting in intestinal inflammation in grass carp (Yao et al., 2022). Also, diets that include tannins reduce feed utilization and immunity and damage the hepatic structures of carp (Liu et al., 2021b). Additionally, a diet supplemented with tannins significantly decreases milk urea nitrogen (MUN) content in lactating dairy cows (Zhang et al., 2019). Moreover, the presence of tannins in animal diets is associated with reduced feed conversion efficiency and retard growth (Jansman, 1993). Also, tannins have a negative impact on vitamin and mineral metabolism. For example, feeding rats with 3.2% tannic acid reduced vitamin A and cobalamin absorption (Jansman, 1993). These findings highlight the challenges of incorporating tannins into animal feed without compromising nutritional quality.

### 4.4 Sinapines

Sinapine, a choline ester of sinapic acid, is a major phenolic component in RSMs, which ranges from 1%-2.5% under different Canadian conditions (Mueller et al., 1978). About 80% of total phenols are constituted by sinapines (Krygier et al., 1982). Sinapine results in a bitter taste, off-flavors, dark color, and astringency to the RSM, which reduces its palatability (Josefsson and Uppstrom, 1976). Sinapines are not subjected to hydrolytic cleavages in plants and are catabolized only during germination (Sosulski, 1979). However, alkali hydrolysis extraction yields free phenolic acid and sinapic acid, which is known for its bitter taste (Durkee and Thivierge, 1975), and sinapine is one of the major phenolic components in rapeseed hulls (Durkee and Thivierge, 1975).

Previous studies have confirmed the presence of fishy taint in brown eggs when the chickens are fed RSM (Goh et al., 1979). In the gut, sinapine is hydrolyzed by microbes, releasing sinapic acid and trimethylamine. Sinapic acid reduces protein digestibility in RSMs (Qiao and Classen, 2003; Qiao et al., 2008). Trimethylamine is oxidized by trimethylamine oxidase in the liver and kidney and then excreted (Butler et al., 1982). However, birds with low concentrations of trimethylamine oxidase tend to develop a fishy taint in eggs. The reduction of enzyme concentration is related to ANFs such as tannins, glucosinolates, and progoitrin. The breakdown products of tannins and progoitrins, such as goitrogen and soluble tannins, reduce the activity of trimethylamine oxidase, resulting in a foul smell in the eggs. Moreover, liver damage caused by breakdown products of tannins and glucosinolates hinders the possibility of using RSM for poultry.

Thus, ANFs pose severe physiological health impairments in both humans and animals, requiring strict intake limitations. Reducing ANFs is essential to improving RSM quality and establishing its implications in the food industry. This review focuses on different strategies to achieve these goals, such as microbial fermentation, breeding, and biotechnological interventions.

## 5 Strategies to reduce ANFs in rapeseed byproducts

**Figure 2:**
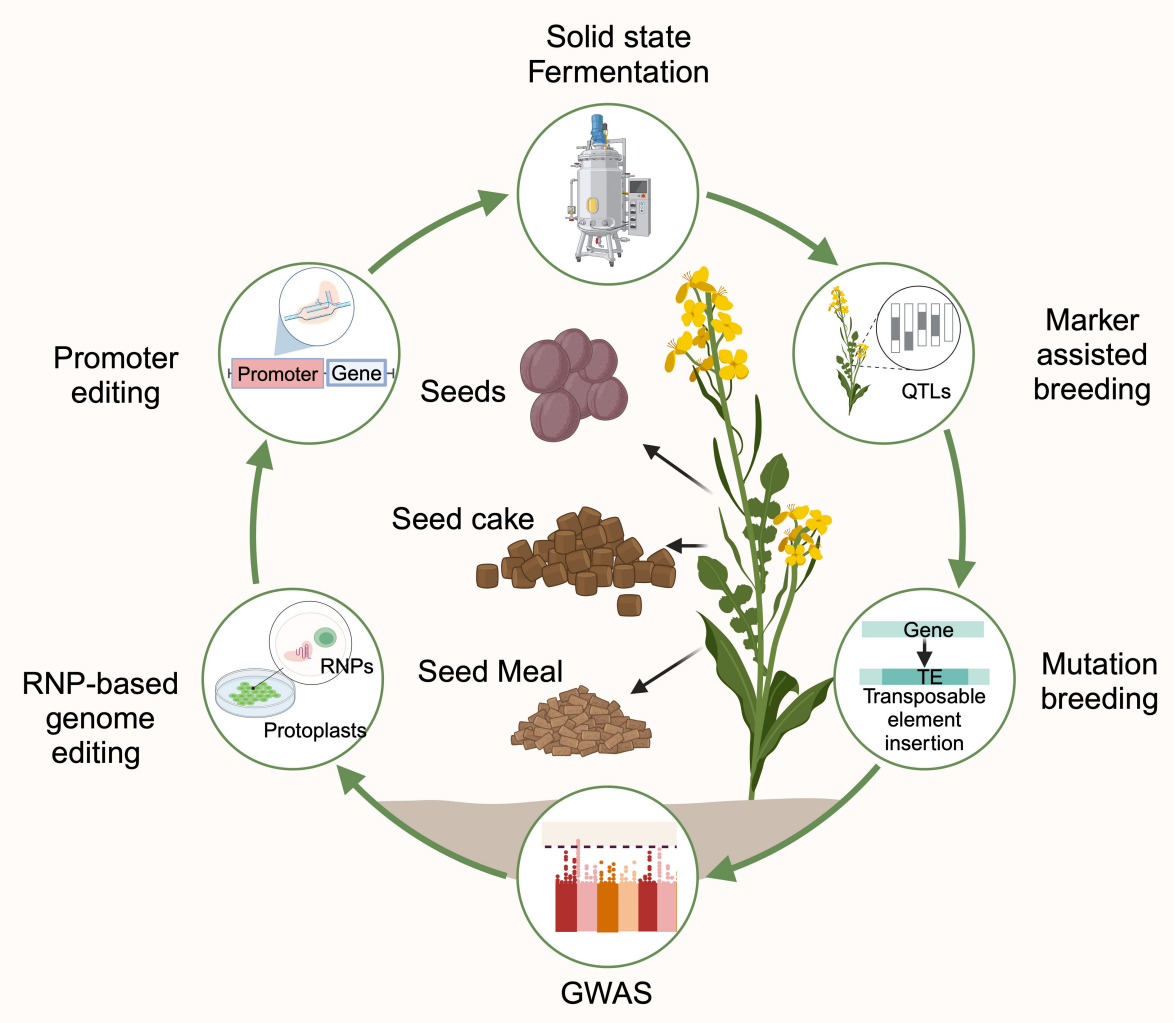
Schematic representation of rapeseed byproducts inclusion in human food and animal feed (Created with BioRender.com).

The quality of rapeseed byproducts can be enhanced by the following two strategies (**Figure 2**):

1. The first strategy focuses on reducing the ANFs in rapeseed byproducts through microbial fermentation to enhance the bioavailability of nutrients and palatability.
2. The second strategy focuses on improving protein content by manipulating protein profiles and reducing ANFs through advanced breeding and biotechnological approaches.

However, improving the protein profile of rapeseed is challenging due to its heritability, association with several nutritional characteristics, and sensitivity to environmental conditions (Wu et al., 2005; Poisson et al., 2019). It is also important to achieve balanced ANF profiles without compromising plant defense mechanisms (Barbehenn and Peter Constabel, 2011; Burow and Halkier, 2017). Reducing the ANFs should be a primary breeding objective to include RSM in the existing food system. Improving the protein profile without reducing ANFs will hinder its implementation in the food chain by affecting its sensory characteristics and acceptance.

### 5.1 Microbial fermentation to improve RSM quality

Various chemical, thermal, mechanical and biological treatments have been investigated to optimize the reduction of ANFs and enhance the nutritional quality of RSM for use as food and feed material. These treatments aim to balance efficiency and feasibility on a large scale without compromising benefits. Physical treatments include thermal, radiation, hulling and others. Thermal processing effectively reduces ANFs but negatively impacts protein content and digestibility (Jensen et al., 1995; Hansen et al., 2017; Tayyab et al., 2017). Irradiating rapeseed cake (RC) using gamma radiation is reported to reduce the ANFs (Anwar et al., 2015; Xiong et al., 2024). However, biohazards and the cost involved with the process limit its utilization. Hulling reduces the seeds’ fiber content but has no significant effects on all other ANFs (Kracht et al., 2004). Similarly, chemical treatments, including acid or alkali supplemented with alcoholic solvents, reduce the ANFs in the meal. However, chemical treatments adversely affect the protein structure and digestibility.

Microbial fermentation involving bacteria, fungi and yeast is a well-established and effective natural method for reducing ANFs while improving the nutritional quality of rapeseed (Vig and Walia, 2001; Wang et al., 2019; Boroojeni et al., 2022; Zaworska-Zakrzewska et al., 2023) (**Table 6**). Solid state fermentation (SSF), which occurs on the surface of water-insoluble materials with or without soluble nutrients and without free-flowing liquid, offers several advantages over other methods. These include low energy consumption, mild reaction conditions (Reddy et al., 2008) and reduced water content in the final product (Xie et al., 2015). The lower moisture content in SSF compared to submerged liquid fermentation facilitates a more accessible commercialization (Chen, 2013).

**Table 6:** Different microbes utilized in improving the quality of rapeseed meal.

S.no	Microorganism	Condition	Effects	Reference
1	*Pleurotus ostreatus*	Solid state fermentation	Increased protein content by 19.2% and improved methionine, isoleucine, tyrosine and phenylalanine, content of the meal. Reduced glucosinolates, sinapine, and phytate content by 96.7%, 95.5%, and 55.0%, respectively.	(Heidari et al., 2024)
2	*Rhizopus oryzae*, *Mucor indicus*, *Trichoderma reesei*	Solid state fermentation	*T. reesei* and *R. oryzae* reduced the structural carbohydrates in CM by 26.5% and 14.5%, respectively. Supplying urea lowered the structural carbohydrates degradation. *T. reesei* and *M. indicus* degraded sinapine in both urea-treated and non-urea treated cases.	(He et al., 2023)
3	*Lactobacillus (L. reuteri* L45, *L. plantarum* L47, and *L. johnsonii* L63) and enzymes (cellulase and pectinase)	Enzymatic hydrolysis (a mixture of 10 FPU/g cellulase and 10 U/g pectinase) for 28 h followed by *Lactobacillus* fermentation for RSM for 48 h	Rapeseed meal treated with enzymes and *Lactobacillus* lowered glucosinolate levels and improved crude protein levels. Enzymatic hydrolysis could promote *Lactobacillus* fermentation by providing reducing sugars.	(Zhu et al., 2021)
4	*Aureobasidium pullulans* (NRRL-58522), *A. pullulans* (NRRL-42023), *A. pullulans* (NRRL-Y-2311-1), *Trichoderma reesei* (NRRL-3653), *Fusarium venenatum* (NRRL-26139), *Pichia kudriavzevii*, *Mucor circinelloides*	Solid state fermentation	The protein content was increased and glucosinolate content was reduced. *T. reesei* was best among the other microbes.	(Croat et al., 2016)
5	*Rhizopus oligosporus*	Solid state fermentation	Glucosinolates, thiooxazolidones, phytic acid and crude fibre were decreased by 43.1%, 34%, 42.4% and 25.5%, respectively.	(Vig and Walia, 2001)
6	*Lichtheimia* sp. JN3C, *Aspergillus terreus*, *Candida tropicalis* CICIM Y0079(T)	Solid state fermentation	Protein levels increased by 27% and glucosinolate levels were decreased by 96%.	(Wang et al., 2012)
7	*Aspergillus niger*	Solid state fermentation 70% rapeseed cake and 30% wheat bran	76.89% degradation of glucosinolates.	(Shi et al., 2015)
8	*Saccharomyces cerevisiae, Saccharomyces boulardii*	Solid state fermentation	Degradation of glucosinolates and 3-butyl isothiocyanate, by 51.60%-66.04% and 55.21%-63.39%, respectively.	(Vlassa et al., 2022)
9	*Bacillus licheniformis* (1.0813), Yeast (ACCC20060), *Lactobacillus* (ACCC10637)	Solid state fermentation	85.02% degradation of glucosinolates. Significant decrease in NDF contents. Rapeseed peptide content increased from 11.9 mg/g to 66.0 mg/g after fermentation.	(Wang et al., 2019)

SSF is more commonly applied to RSM for ANFs reduction than liquid-phase fermentation. It prevents strong drying after fermentation, which often occurs in high-pressure and heat sterilization, leading to rapeseed protein denaturation and reduced conversion efficiency (He et al., 2014). Fermentation processes enhance protein content, vitamin and mineral levels, and digestibility while improving sensory profiles and nutrient utilization (He et al., 2012; Shi et al., 2016). Furthermore, fermentation increases proteolytic enzyme activity, promoting the hydrolysis of ANFs into low molecular weight peptides and detoxifying the RSM (He et al., 2012). These low molecular weight peptides derived from rapeseed exhibit improved protein functional properties, antioxidant activity, and other beneficial biological activities (Pinterits and Arntfield, 2008).

Several studies have explored the reduction of glucosinolates, phytic acid, and other ANFs through SSF (Wang et al., 2012). Due to their broad metabolic activities, fungal fermentations are particularly promising for reducing ANF components and biofortifying substrates with positive health effects (Wang et al., 2012; Shi et al., 2015). The filamentous fungus *Aspergillus*, recognized as a safe microorganism (GRAS- Generally Recognized as Safe), produces various degradation enzymes, including protease, phytase and hydrolases, which effectively degrade glucosinolates, phytic acid and other ANF components (El-Batal and Karem, 2001; Shi et al., 2015). Similarly, *Rhizopus oligosporus*, a GRAS organism commonly used as a “tempeh” starter, degrades glucosinolates and other phenolic compounds (Bau et al., 1994; Lücke et al., 2019). *R. oligosporus* has been shown to reduce glucosinolate levels by 47%, lignin and other polyphenolics by 30%, phytic acid by 42.4%, and total fiber content by 25.5% while simultaneously increasing the nitrogen and protein content of RSM (Lomascolo et al., 2012). Members of the *Aspergillus* genus are also widely recognized for their ability to degrade phytic acid in rapeseed during SSF (Al-Asheh and Duvnjak, 1995; Ebune et al., 1995a; Ebune et al., 1995b). In addition, white rot fungi, such as *Pleurotus* spp., commonly known as oyster mushrooms, are effective at degrading secondary metabolites. They have been reported to reduce sinapic acid (sinapines) by up to 99%.

Bacterial fermentation is extensively applied to degrade ANFs and enhance sensory properties in food products. Among the fermentative bacteria, *Lactobacillus* species are considered safe (GRAS) organisms and the most popular and traditional fermentation bacteria used as probiotics. These bacteria produce lactic acid, which lowers the pH of the substrate, thereby enhancing flavor and nutritional profile while inhibiting the growth of pathogens. *Lactobacillus salivarious* fermentation has been shown to reduce glucosinolate and fiber content in rapeseed (Aljuobori et al., 2014). Similarly, *L. plantarum* degrades the glucosinolates by up to 80% and reduces phytic acid in RSMs by secretion of extracellular enzymes. This process reduces the ANFs and enhances the soluble protein content and bioavailability, thereby improving the overall nutritional quality of the RSM (Chen et al., 2024).

Similarly, yeast fermentation effectively reduces the content of ANFs and improves nutrient quality. Various *Saccharomyces* species, such as *Saccharomyces cariocanus*, *Saccharomyces cerevisiae*, *Saccharomyces boulardii* and *Wickerhamomyces anomalus*, have been extensively explored in yeast fermentation to reduce ANFs and enhance the nutritional properties of rapeseed (Vlassa et al., 2022). Fermentation with *S. cerevisiae* or *S. boulardii* has been shown to minimize glucosinolate content by 51%-66%. Additionally, yeast fermentation decreases the carbohydrates, phenolic acids, organic acids, sinapic acid, tannins and phytic acid contents while increasing the protein content of RSM (Bau et al., 1994; Vlassa et al., 2022).

Co-inoculation with specific bacterial species such as *Lactobacillus* and *Bacillus* (including *Bacillus cereus*, *Bacillus subtilis*, and *Bacillus licheniformis*) has proven to be an efficient method for reducing glucosinolate content and enhancing protein content (Wang et al., 2019). Similarly, the combination of bacteria, fungi, and yeast strains, resulting in a diverse pool of enzymes, has shown superior ANFs degradation capabilities compared to single-strain fermentations (Xu et al., 2012; Yusuf et al., 2021). This mixed-strain approach has significantly improved ANFs degradation efficiency, particularly for glucosinolates, achieving up to 83% reduction (Yusuf et al., 2021).

Despite its benefits, fermentation has several challenges in the food industry. The process is prone to inconsistent product quality due to variations in microbial activity and prolonged fermentation (Zentek and Boroojeni, 2020). This leads to potential contamination and the growth of unwanted pathogens, which introduce health risks and affect product quality (Capozzi et al., 2017). Scaling up fermentation to industrial levels introduces complexities in maintaining consistent microbial activity, impacting the uniformity of the final product. This scaling requires specialized equipment, costly infrastructure, increased operational costs, and stringent environmental controls (Tamang et al., 2020).

Fermented foods must comply with food safety regulations and require continuous monitoring and testing to ensure safety and quality. The sensory changes fermentation induces can sometimes produce undesirable characteristics, affecting consumer acceptance (Marco et al., 2017). Therefore, addressing these challenges is crucial for successful fermentation processes.

Hence, microbial fermentation is a practical, robust, and versatile method for reducing ANFs in rapeseed. By strategically employing specific microorganisms in monoculture or combination with their fermentation techniques, it is possible to enhance rapeseed’s nutritional profile, sensory properties, and usability. This approach effectively addresses the challenges posed by ANFs. It adds value to rapeseed as a sustainable and nutritious resource, making it a valuable ingredient in both food and feed industries.

### 5.2 Classical breeding

*B. napus*, commonly known as rapeseed (2n = 38, AACC), is an allotetraploid crop from natural interspecific hybridization between *B. rapa* (2n = 20, AA) and *B. oleracea* (2n = 18, CC). Like other crops, the primary breeding objective in rapeseed is to increase oil yield. However, the high erucic acid (~40%) and glucosinolate (80 µmol/g) content hindered the use of rapeseed oil and meal for consumption (Daun, 1986). Intense breeding efforts successfully reduced these contents, and the first low erucic aid and low glucosinolate variety, Tower, was released by Canada and was named a double zero rapeseed variety. Breeding strategies for yield improvement, (a)biotic stress resistance, and nutritional improvement were primarily prioritized. Recently, the focus has shifted towards meal quality by modifying their protein profiles, which have been given importance for their use in the food industry (Wanasundara et al., 2017).

Selecting yellow seed coats can effectively reduce the fiber and tannin contents while improving the protein content of rapeseed. Yellow-seeded genotypes have thinner seed coats, lower hull content, and large embryos with higher oil and protein content in the seeds (Bell and Shires, 1982). Pro-anthocyanidins and tannins determine the seed coat color; darker seed coat colors are associated with high tannin content, which reduces the meal digestibility (Bell and Shires, 1982). Moreover, brown and black-seeded varieties have higher fiber content than yellow-seeded varieties, reducing meal digestibility (Shirzadegan and Röbbelen, 1985). The lack of natural yellow-seeded types in *B. napus* lead to development of varieties by interspecific crosses involving *B. rapa*, *B. juncea, B. carinata* and *B. campestris* spp. *alboglabra, B.oleraceae* (Rashid et al., 1994; Meng et al., 1998; Zhang et al., 2004; Rahman, 2008). The progenies from these interspecific crosses were subjected to further breeding techniques like pedigree and backcrossing to develop the yellow-seeded rapeseed lines, which are subsequently developed into varieties.

#### 5.2.1 Inheritance of seed coat color and seed storage proteins of *Brassica*

Understanding the genetic control behind the seed color is essential for breeding rapeseed genotypes with increased protein and reduced fiber contents. Two independent dominant genes, *Br1* and *Br3*, are responsible for seed coat color. Dominance at the *Br1* locus results in brown seeds, while the dominant *Br3* locus with a homozygous recessive *Br1* locus results in yellow-brown seeds. Both loci in a homozygous recessive condition produce yellow seeds (Stringam, 1980).

In *B. napus*, three genes are responsible for the seed coat color expression (Rahman et al., 2010). The seed coat colors are black to dark brown, light brown, dark yellow, light yellow and yellow. The genes *BSNap1 and BSNap2* control black/brown seed coat color, while the gene *DKYSNap3* influences dark/light yellow seed coat color (Rahman et al., 2010). Homozygous recessive condition across all three genes results in yellow seeds. Studies on the seed coat color inheritance in F_2_, BC_1_ (Back Cross 1) and the DH (Double Haploid) population revealed the epistatic interactions between the dominant gene for yellow seed color and two independently segregating dominant genes for black color seeds (Liu et al., 2008).

Quantitative trait loci (QTLs) mapping identified two QTLs responsible for seed coat color, which accounted for 46% and 30.9% variation (Liu et al., 2006). Further discoveries of QTLs for seed coat color, protein, and fiber contents concluded that many genes control these traits and are highly influenced by the environment. For example, temperatures can affect the yellow and black-seeded rapeseed varieties. High temperatures might disrupt pigment synthesis, resulting in yellow-seeded genotypes (Deynze et al., 1993).

The discovery of QTLs significantly affects marker-assisted selections (MAS) and genomic selections. Identification of molecular markers associated with QTL of desirable traits facilitates MAS. This enables early selection, pyramiding favorable QTLs, and reduces the duration of the breeding cycle to get favorable phenotypes with improved performance. However, the QTL analysis for seed quality traits in rapeseed requires trials in multiple environments to minimize the environmental influence and determine stable QTLs across environments. Only upon validation of such major and consensus QTLs, they can be recruited in MAS programs.

As mentioned above, SSPs have immense potential in the food processing industry. However, breeding for favorable genotypes requires understanding the trait’s genetic basis. SSP content is a quantitative trait that involves complex gene regulation and biosynthesis. The SSPs, cruciferin and napin are coded by multiple genes, particularly 9-12 genes involved in the cruciferin biosynthesis (Wanasundara, 2011), and 10 genes are responsible for the napin biosynthesis (Josefsson et al., 1987). SSP content within the seed also depends on post-translational processes and the transfer of assembled SSP into storage vacuoles (So and Duncan, 2021). Several QTLs are associated with SSP content. QTLs Nap-1, Nap-2, Nap-3, Cru-1, Cru-2, Nap/Cru-1, Nap/Cru-2, and Nap/Cru-3 were identified in the double haploid populations of winter oilseed rape (Schatzki et al., 2014). Moreover, transcription factors such as *Abscisic acid insensitive 3* (*ABI3*), *Fusca 3* (*FUS3*), *Leafy Cotyledon 1* (*LEC1)* and *Leafy Cotyledon 2 (LEC2)* are involved in the SSP regulation (Vicient et al., 2000; Kroj et al., 2003; So and Duncan, 2021). Hence, understanding the genetic control and regulatory mechanisms behind SSPs is crucial for modern breeding strategies to enhance SSP content for improved food processing applications.

#### 5.2.2 Mutation breeding

Classical mutation breeding techniques have often focused on increasing rapeseed oil yield and quality (Röbbelen, 1990). Nonetheless, mutation breeding also has immense potential for reducing ANFs (Harloff et al., 2012) and increasing the protein fractions in RSM. Double haploid mutant lines produced from seeds or microspores are a promising option for producing successful mutations. The National Research Council of Canada (NRC) utilized this approach and recently reported a mutant line with a 7% increase in protein content (National Research Council Canada, 2023; available at: https://nrc.canada.ca/en/stories/boosting-canola-crop-value-mutation-breeding).

Similarly, transposon element (TE) insertions provide a biological means of inducing plant mutations. Transposons are mobile elements in plants that were once considered junk DNA and are now a valuable tool for breeders to create favorable plant variations. TE insertions in the coding regions will lead to loss-of-function mutations. In contrast, insertion in intron regions can result in new isoforms through exonization, alternative splicing, truncation, or a combination of these mechanisms (Dubin et al., 2018). Moreover, TE insertions can potentially disrupt enhancers or regulatory promoter elements outside the transcribing regions, thereby altering the transcriptional level (Dubin et al., 2018). TE activation can be achieved through toxins that inhibit DNA methylation, and stress-mediated TE activation has also been reported in different crops (Ito, 2022), for example, *in vitro* regeneration stress results in TE element activation in rice (Hirochika et al., 1996) and tobacco (Hirochika, 1993). However, TE-mediated mutagenesis requires robust methods to induce TE activation and transgenerational inheritance of TE insertions for further breeding implications (Kirov, 2023).

TE insertions in rapeseed have improved silique length and seed weight and reduced pod shattering. CACTA-like TE (3.9 kb) inserted in the upstream of *BnaA9.CYP78A9* increased the expression of the loci, resulting in elongated siliques (Shi et al., 2019). Similarly, long-term repeat retrotransposon insertion (4803 bp) in the promotor of *BnSHP1.A9* repressed its expression and conferred resistance to pod shattering (Liu et al., 2019). However, such transposons-mediated mutagenic studies have yet to be conducted to improve seed meal quality traits.

Mutation breeding could be essential as it avoids legislative pressure and regulations. Despite its potential, mutation breeding faces challenges due to a low frequency of mutation occurrence, unspecific mutations causing undesirable mutations, and safety hazards associated with the use of dangerous physical and chemical mutagenic agents.

### 5.3 Molecular breeding strategies

Modern breeding techniques offer numerous advantages, such as improved efficiency in breeding, precision to improve the target traits and reduced breeding cycles. The novel tools of biotechnology and genomics have transformed the approaches for breeding complex characteristics that are quantitative, pleiotropic and sensitive to environmental factors, which were challenging for the traditional breeding approaches to address. The introduction of molecular markers made the first significant leap in breeding strategies. PCR-based DNA markers have become integral in almost all areas, like pre-breeding to broaden the genetic base, aid in effective selections, introgression of desired characters, determining the heterotic potentials, and improving the quantitative traits.

Biparental mapping populations, which include F2 populations, recombinant inbred lines (RILs), double haploid lines (DH), backcross populations (BC), and near-isogenic lines (NILs), are developed from crossing two parents. The greater the contrast traits expressed by the parents, the more variations can be retrieved in the mapping populations. These populations are generally used for QTL detections, and the size of the population also determines the reliability of the identified QTLs. Several mapping populations have been created to detect QTLs for agronomic and seed traits in rapeseed (Chen et al., 2007, Chen et al., 2010; Fattahi et al., 2018; Yusuf and Möllers, 2024).

However, the time and cost of generating and maintaining biparental populations far exceed those required for association mapping (AM). Biparental mapping populations consider only the allelic variations between two parents. This narrows the allelic variations and in turn, limits the QTL detections for trait improvement (Singh et al., 2015). This limitation highlights the need for alternative methods like AM, which can incorporate a broader genetic base and provide more comprehensive insights into trait variation and improvement.

#### 5.3.1 Genome-wide association studies

Genomics in breeding enables the exploration of various advanced tools to obtain accurate population genotypic data. Genome sequencing technologies provide high throughput genome sequencing data with high accuracy and reliability. The recent advances in the discovery of single-nucleotide polymorphism (SNP) markers through genome-wide association studies (GWAS) improved the efficiency of correlating the phenotypic and genotypic data and selection efficiency for precision breeding. *Brassica napus* 60K Illumina Infinium™ SNP array allows multiplex SNP genotyping, enabling simultaneous surveys of thousands of SNPs and increasing the efficiency of the genotypic data (Wang et al., 2014; Mason et al., 2017; Zhou et al., 2021).

AM approach facilitates the identification of markers closely linked to the genes of interest. Unlike linkage mapping, which relies on recombination events between the gene and the marker in a bi-parental cross, AM utilizes all the recombination events that would have happened in the past in the population (Singh and Singh, 2015). The post-GWAS era offers new approaches for processing GWAS data to extract meaningful information by identifying and functionally characterizing candidate genes using bioinformatics and reverse genetics approaches. Targeting-induced local lesions in genomes (TILLING) approaches functionally characterize candidate genes identified via GWAS in mutagenized populations. The Eco-TILLING approach is used in germplasms or natural populations (Gupta et al., 2019). GWAS studies have been successfully utilized for several traits in rapeseed, including disease resistance, flowering-related traits, salt tolerance, aluminum tolerance and silique length (Helal et al., 2021; Roy et al., 2021; Wang et al., 2021; Zhang et al., 2022; Zhou et al., 2022; Starosta et al., 2024; Zhang et al., 2024). Similarly, GWAS has been successfully applied to rapeseed for various seed quality traits like fiber traits, ANFs, oil, and amino acid content (Wang et al., 2015; Wei et al., 2019; Bhinder et al., 2022; Tan et al., 2022; Zhao et al., 2022). However, studies focusing on seed storage proteins seem to be lacking (So and Duncan, 2021). Even though GWAS has identified many QTLs for essential traits in rapeseed, this technique faces challenges such as poor reproducibility and false positives, indicating the need for further improvement in this crop.

#### 5.3.2 Marker-assisted selection

Traditional breeding focuses on phenotypic selection for genetic improvement. However, recent developments in genomics led to the identification of several QTLs useful for marker-assisted selection (MAS). MAS facilitates an indirect selection based on markers linked to the target traits. These markers are highly reliable, unaffected by the environment, and non-specific to plant growth stages, promoting early selection and reducing the breeding cycle. Combining MAS with speed breeding techniques can reduce the breeding cycles, thus promoting the development of new varieties in short periods. However, focusing solely on genetic composition might mislead the selection of agronomically poor phenotypes. The phenotypic selection combined with MAS is always preferred to achieve all the agronomic and quality traits.

The trait of yellow seeds was confirmed to be quantitative after intense research by various scientists (Liu et al., 2006; Fu et al., 2007; Chao et al., 2022a). The QTL discovery for fiber content and its correlation with the yellow seeds paved the way for genetic manipulation (Badani et al., 2006). The fiber content of the seeds compromises acid detergent fiber (ADF), neutral detergent fiber (NDF), and acid detergent lignin (ADL). Hemicellulose, cellulose, and lignin predominate in NDF, while the ADF comprises cellulose and lignin. ADL represents the nondigestible lignins in the seeds (Behnke et al., 2018). Altering these fractions could be beneficial for animal husbandry.

Different DNA-based molecular marker systems have been used for MAS to improve these quality traits. The yellow-seeded trait associated with 8 RAPD markers co-segregated with a single major gene (*Pigment 1*) responsible for the yellow seed coat color trait (Somers et al., 2001). Other markers, including restricted fragment length polymorphism (RFLP), amplified fragment polymorphism (AFLP), cleaved amplified polymorphic sequences (CAPS), sequence related amplified polymorphism (SRAP), sequence characterized amplified region (SCAR), and simple sequence repeat (SSR) markers (Deynze et al., 1995; Liu et al., 2006; Rahman et al., 2010; Zhang et al., 2010b; Huang et al., 2016) were also identified for breeding yellow seeds and low fiber content. Pleiotropy arises when a single gene or genetic locus affects multiple phenotypic traits. Pleiotropic effects between the target traits are advantageous as they facilitate breeding. Selecting one QTL might give the desired phenotype for the other targeted trait.

The QTL analysis by various scientists (**Table 7**) clearly states that QTL at A09 is a major QTL for seed color and fiber traits (Liu et al., 2013; Miao et al., 2019; Chao et al., 2022a). The QTL at C05 is a major QTL for fiber and protein traits (Yusuf and Möllers, 2024). Focusing on these loci could provide desirable phenotypes. An apparent pleiotropic effect exists between seed fiber traits, plant height, and protein content (Wurschum et al., 2012; Liu et al., 2013; Miao et al., 2019). Additionally, a negative correlation exists between oil content and fiber characteristics, indicating that reducing the fiber content could improve the oil content. However, enhancing protein profiles will decrease the oil content due to their negative correlation (Yu et al., 2016; Chao et al., 2017, Yusuf and Möllers, 2024).

**Table 7:** Major QTLs for the improvement of the meal quality traits.

Trait	Markers	Population	No of QTLs	Chromosome	Comments	Citation
NDF	SNP markers	Double haploid population of Adriana x SGEDH13	4	A01, C01, C04, C05	C05 has the major QTL for NDF, ADF, LC, HC, OC+SPC, PidM. Oil content is negatively correlated to SPC. SPC negatively correlated to ADF, NDF and positively correlated to GC. GC negatively correlated to NDF.	(Yusuf and Möllers, 2024)
ADF			6	A01, A04, A07, A10, C01, C05		
LC			4	A04, A07, A10, C05		
HC			3	C01, C04, C05		
CC			3	A01, A07, C01		
SPC			3	A02, A07, C01		
OC+SPC			2	C03, C05		
PidM			5	A07, A10, C01, C03, C05		
GC	SNP markers	Double haploid population of Adriana x Zheyou 50	2	A02, A09		
SC	9164 SNP markers	RILs population from GH06 x P174	1	A09	Pleiotropic effects between CC and HC QTLs at A09.	(Liu et al., 2013)
ADL			2	A09, C05		
CC			4	A08, A09, C02, C03		
HC			3	A03, A06, A09		
PC	253 SNP markers	391 doubled haploids lines derived from nine crosses among 10 parental lines	2	A07, C03	Pleiotropic effects between plant height and PC QTLs at A7.	(Wurschum et al., 2012)
GC			2	A03, A07	Negative correlation between OC and GC.	
LC	SNP, SSR, STS markers	Doubled haploid population derived from Ken-C8 x N53-2	29	A02, A03, A06, A07, A09, A10, C05, C09	Negative correlation between LC, CC, HC to OC. A09 is the major QTL for fibre traits. 13 pleiotropic unique QTL for seed fibre components and OC were also reported.	(Miao et al., 2019)
CC			35	A04, A08, A09, A10, C01, C03, C05, C06		
HC			21	A06, A07, A09, C02, C04, C06, C08		
SC	3207 SNP markers	Double haploid population derived from N53-2 x Ken-C8	10	A09, A10, C01, C03, C05, C08	A major QTL, *cqSC-A09*, on chromosome A09 was identified. *cqSC-A09* controls the SC and fibre traits. Yellow seed negatively correlated to fibre traits.	(Chao et al., 2022a)
ADF	265 SSR, 17 SNP, 38 GEM markers	Double haploid population	2	N16, N9	QTLs for three fibre traits (ADF, ADL and NDF) and seed colour- whiteness index (WIE) were mapped on N9. Strong negative correlation between PC and OC.	(Yu et al., 2016)
ADL			2	N19, N9		
NDF			4	N4, N9, N13, N16		
PC			3	N16, N18, N3		
SC	420 SSR, RAPD, SRAP	RIL1 GH06 x Zhongyou 821	12	N5, N9, N6	*TT10* as a candidate gene for SC.	(Fu et al., 2007)
		RIL 2 GH06 x Youyan 2	7	N4, N12, N16, N8		
PC	3207 SNP markers	Double haploid population derived from Ken-C8 x N53-2	38	A02, A03, A04, A07, A09, C01, C03, C05, C06, C07, C08, C09	High genetically negative correlation between OC and PC.	(Chao et al., 2017)
GC	SSR, STS, SRAP, SNP markers	Double haploid population derived from N53-2 x Ken-C8	59	A01, A06, A07, A09, A10, C02, C03, C07, C08, C09	Chromosomes A09, C02, C07 and C09 contained QTL hotspot regions (QTL-HRs) for seed GC.	(Chao et al., 2022b)
GC	SNP, SSR markers	Double haploid population derived from ZS11 x 195-14A	29	A1, A2, A3, A4, A5, A6, A7, C2	*cqSGC-C2* was identified as major QTL.	(Zhou et al., 2021)

NDF, Neutral Detergent Fibre; ADF, Acid Detergent Fibre; LC, Lignin Content; HC, Hemicellulose Content; CC, Cellulose Content; SPC, Seed Protein Content; OC, Oil Content; PidM, Protein Content Defatted In Meal; GC, Glucosinolate Content; SC, Seed Color; PC, Protein Content; RIL, Recombinant Inbred Lines; ADL, Acid Detergent Lignin.

### 5.4 Genome editing as an advanced biotechnological intervention

Genome editing has revolutionized breeding strategies by focusing on molecular manipulation to achieve desirable plant ideotypes. This technology offers promising solutions for important breeding objectives such as climate-resilient crop breeding, nutritional improvement, and biotic and abiotic stress tolerance within a short time compared to conventional breeding techniques (Dhugga, 2022). The precision and specificity of the editing tool are essential for successful genetic manipulation. The discovery of CRISPR (clustered regularly interspaced short palindromic repeats) Cas9 (CRISPR-associated protein 9) has revolutionized the field of agriculture with its high specificity and precision. CRISPR/Cas9 technology originated from type II CRISPR/Cas systems, an adaptive immunity mechanism in bacteria, providing defense against viruses and plasmids (Doudna and Charpentier, 2014). CRISPR/Cas9 uses a guide RNA sequence targeting specific DNA sequences and endonuclease Cas9 cleaving the targeted site’s DNA. The protospacer adjacent motif (PAM) sequence adjacent to the single guide RNA (sgRNA) is critical for DNA binding by Cas9. Without the PAM sequence, sgRNA, even if fully complementary to the target sequences, is not recognized by the Cas9 protein (Doudna and Charpentier, 2014).

#### 5.4.1 Ribonucleoprotein-based genome editing

The delivery of the CRISPR/Cas9 system into the host requires a vector. *Agrobacterium-*mediated delivery of sgRNA and Cas9 protein is widely deployed in genome editing. *Agrobacterium tumefaciens*, a gram-negative soil bacterium, is responsible for causing gall growth in plants. It possesses a Ti plasmid, which makes it a perfect biological tool for vector constructs. The T-DNA region of a Ti plasmid is approximately 15–30 kb in size (Rahman et al., 2023). The DNA insert size is also limited in this type of gene transfer, thus limiting the possibility of the multiplexing techniques. The transfection process is mediated by inserting the gene of interest by replacing the T-DNA region and co-cultivating the bacteria with the explant. Successful *Agrobacterium*-mediated genome editing was done in rapeseed, targeting seed coat color and tannins (Cheng et al., 2023; Li et al., 2024).

**Figure 3:**
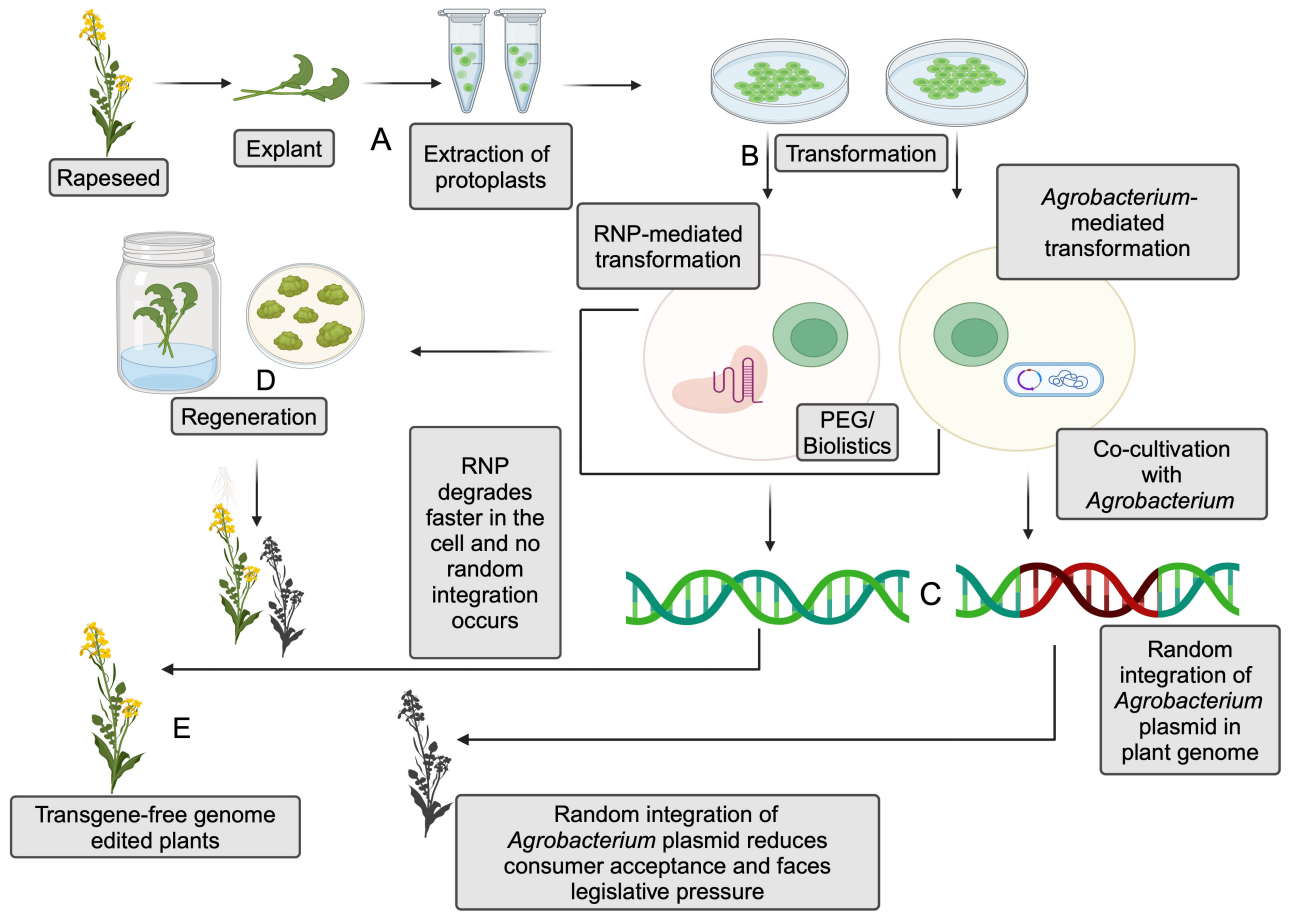
Comparison between ribonucleoprotein (RNP)-mediated and *Agrobacterium*-mediated genome editing in the protoplasts. **(A)** Extraction of protoplasts from explant. **(B)** Transformation of protoplasts with RNPs or *Agrobacterium*- mediated transformation. Polyethylene Glycol (PEG) or biolistics are utilized to achieve transformation via RNP, and co-cultivation is generally utilized for *Agrobacterium*-mediated transformation. **(C)** Random integration of plasmid DNA in *Agrobacterium*-mediated transformation and absence of such integration in RNP-mediated transformation. **(D)** Regeneration of the plants after transformation. **(E)** Transgene-free genome-editing plants are obtained from the regenerated plants (Created with BioRender.com).

However, transformation efficiency highly depends on the transformation protocols, the nature of the explant, co-cultivation periods, and the markers used in the transformation (Rahman et al., 2023). The random integration of T-DNA from bacterial plasmids into the host raises regulatory concerns, affecting the marketability of the products due to reduced consumer acceptance (**Figure 3**). T-DNA components such as Cas9, gRNA, and selection markers can be effectively removed by selfing or backcrossing with wild-type to obtain edited progeny that is free of foreign DNA.

Another promising alternative for eliminating the foreign DNA integration with the host genome is RNPs, which ensures transgene-free genome editing (**Figure 3**). This technique eliminates the need for a vector to deliver the sgRNA sequence and the protein while still being able to manipulate the DNA. Unlike *Agrobacterium*-mediated CRISPR/Cas9 editing, RNP-mediated editing contains Cas9 enzyme coupled with guide-RNA. The first step in RNP-mediated editing is isolating the protoplasts, followed by the transfection process, usually using polyethylene glycol (PEG) electrophoresis or biolistics for RNP delivery. The protoplasts lack cell walls, thereby facilitating the entry of RNPs into the host cells. The use of RNP complexes can reduce off-target effects and the risk of random DNA integration compared to plasmid-based delivery methods. This is due to the transient nature of RNPs in cells, which limits the time for unintended editing (Woo et al., 2015). However, carefully selecting guide RNAs can further minimize off-target effects in both *Agrobacterium*-mediated and RNP-based gene editing (Young et al., 2019).

Successful RNP-mediated genome editing using the biolistic approach has been shown in various crops (Svitashev et al., 2016; Liang et al., 2017; Banakar et al., 2020). Plasmid and RNP-based gene editing using protoplasts has been successfully demonstrated in rapeseed (Li et al., 2021; Sidorov et al., 2022). However, genomic instability (change in ploidy level due to endoreduplication and aneuploidy) associated with protoplast regeneration has been reported in various crops (Newell et al., 1984; Olin-Fatih, 1996; Sheng et al., 2011; Fossi et al., 2019).

#### 5.4.2 Promoter editing

**Figure 4:**
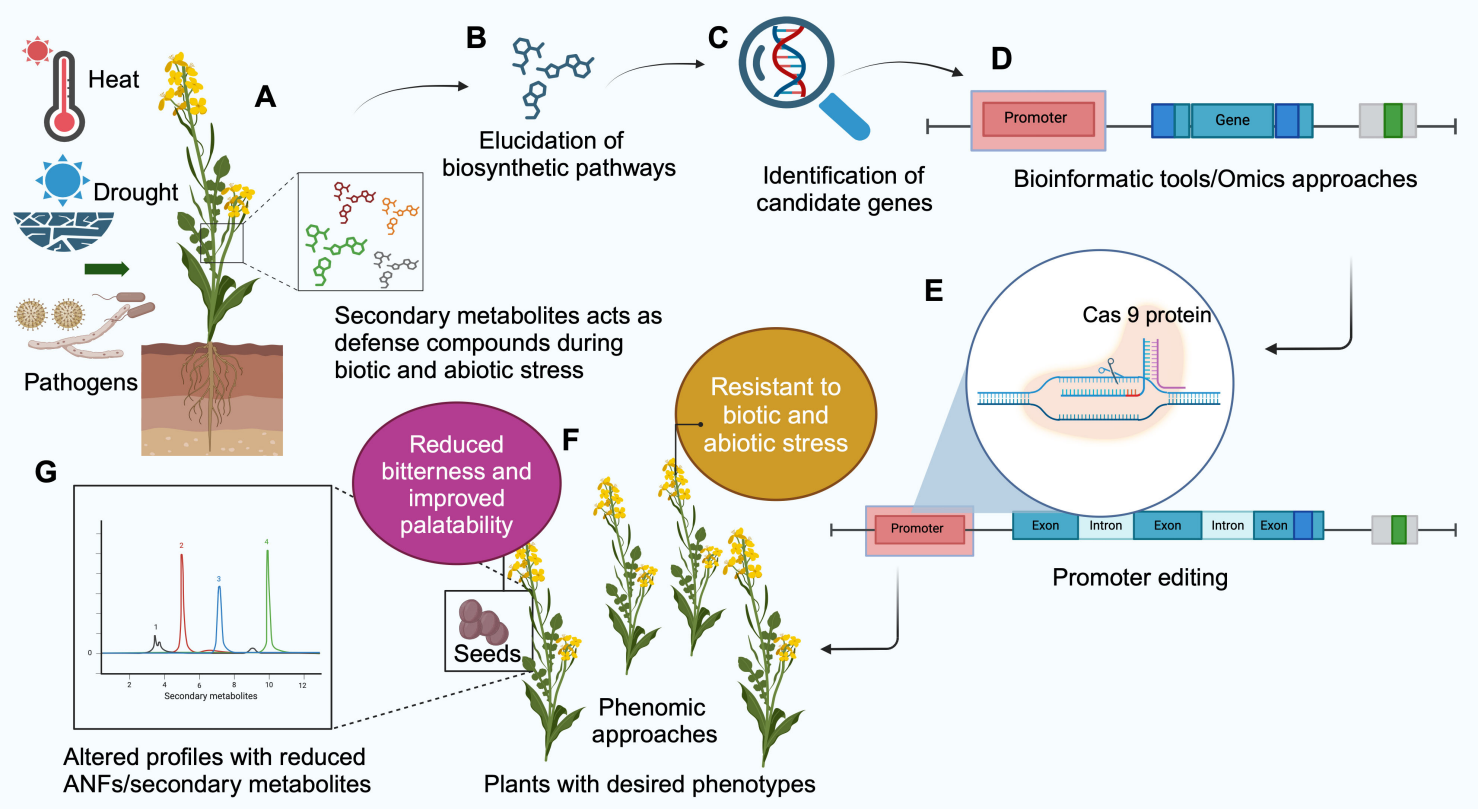
Schematic representation of promoter editing to alter the secondary metabolite/antinutritional factor (ANF) profile in plants. **(A)** Secondary metabolites protect plants during biotic and abiotic stresses. Knocking out the biosynthesis breaks the plant defense against such stress. Promoter editing begins with **(B)** Study of biosynthesis pathway of the metabolite, **(C)** Discovering the genes involved in biosynthesis. **(D)** Identifying the gene sequences of the promoter using bioinformatics tools. **(E)** Promoter region is edited using CRISPR/Cas9 technique. **(F)** The edited plants are subjected to phenomics approaches to identify desired phenotypes with reduced secondary metabolites/ANF without compromising the defense abilities of the plants. **(G)** Metabolomics approaches such as High Performance Liquid Chromatography (HPLC) and Liquid Chromatography-Mass Spectrometry (LCMS) can be used for assessing the profile of metabolites in plants (Created with BioRender.com).

As mentioned before, ANFs play a vital role in plant defense. The complete elimination of ANFs by knocking out the target genes through genome editing may be detrimental to the survival of the plants in some cases. So, reducing their levels instead of eliminating them could keep the defense system intact. Promoter editing (**Figure 4**) could be used to target ANF profiles by altering the gene expression. The promoter regulates the gene expression and is located upstream of the region to be transcribed (Porto et al., 2014). It is a region where RNA polymerase binds to initiate transcription.

Promoter editing begins with assessing the secondary metabolites/ANF compounds that are present in the crop. This is followed by studying the metabolic pathways, enzymes, and compounds involved. Bioinformatics and omics approaches are essential for identifying the genes involved in these pathways and for pre-determining the candidate genes and their promoter regions that must be targeted to alter the ANF profiles. Once the editing is successful, plant phenotyping is essential for the morphological and agronomical assessment of the crop. Screening the phenotypes for disease resistance is also vital to determine that the defense system is not compromised.

Successful promotor editing has been done in various crops (Cui et al., 2020; Huang et al., 2020; Zeng et al., 2020; Liu et al., 2021a; Song et al., 2022b). However, *Agrobacterium*-mediated editing has been followed in promoter editing, which raises biosafety concerns. In this regard, RNP-based promoter editing would be an alternative approach to producing transgene-free mutants to overcome legislative issues.

The candidate genes that could be considered for genome editing to alter the ANF profiles are discussed below (**Table 8**).

**Table 8:** Candidate genes for the improvement of the meal quality traits.

S.NO	Trait	Candidate genes	Gene functions	References
1	Glucosinolates	*GTR1*, *GTR2*, *GTR3*	Glucosinolate Importers	(Nour-Eldin et al., 2012; Jørgensen et al., 2017; Tan et al., 2022; Yusuf and Möllers, 2024)
		*UMAMIT29,* *UMAMIT30,* *UMAMIT31*	Glucosinolate Exporters	(Sanden et al., 2024)
		*MYB28*	Transcription factor	(Gigolashvili et al., 2007; Wang et al., 2018; Wei et al., 2019; Zhou et al., 2021; Yusuf and Möllers, 2024)
		*MYB34*	Transcription factor	(Yusuf and Möllers, 2024)
2	Phytic acid	*PMT5*	Myo-inositol transport	(Bhinder et al., 2022)
		*SAC8*	Phosphatidylinositol dephosphorylation	(Zhong and Ye, 2003)
		*PIP5Ks*	Phosphatidylinositol (4,5)-biphosphate biosynthesis	(Van den Bout and Divecha, 2009)
		*ITPK1,* *ITPK4*	Phytic acid biosynthesis	(Sashidhar et al., 2020b)
3	Sinapine	*BnSGT/UGT84A9*	Sinapine biosynthesis	(Wolfram et al., 2010)
		*BnREF1*	Sinapine biosynthesis	(Mittasch et al., 2013)
4	Lignin	*Quinate hydroxycinnamoyl* *transferase (HCT)*	Lignin biosynthesis	(Liang et al., 2019)
		*Caffeic acid O-methyltransferase* *1 (COMT1)*	Lignin biosynthesis	(Lee et al., 2021)
		*Ferulate-5-hydroxylase*	Lignin biosynthesis	(Cao et al., 2022)
		*BGLU46*	Lignin biosynthesis	(Bhinder et al., 2022)
		*BGLU45*	Lignin biosynthesis	(Bhinder et al., 2022)
		*Cinnamyl-alcohol dehydrogenase*	Lignin biosynthesis	(Bhuiyan et al., 2009; Wang et al., 2015)
		*Phenyl ammonia lyase 4, Cinnamoyl CoA reductase 1*	Lignin biosynthesis	(Wang et al., 2015)
5	Cellulose and Hemi-Cellulose	*Cellulose synthase*	Cellulose biosynthesis	(Hu et al., 2018)
		*Mannose-1-phosphate guanylyltransferase 1*	Cellulose biosynthesis	(Bhinder et al., 2022)
6	Seed storage proteins	*Cruciferin 1*	Encodes cruciferin- storage proteins	(Gacek et al., 2018)
		*Cruciferin 2*		
		*Cruciferin 3*		
		*Amino acid permease1*	Amino acid transporters	(Sanders et al., 2009)
		*Amino acid permease2*	Amino acid transporters	(Zhang et al., 2010a)
		*Cationic amino acid transporter*	Amino acid transporters	(Hammes et al., 2006)
		*Amino acid permease 8*	Amino acid transporters	(Schmidt et al., 2007)

#### 5.4.3 Glucosinolates

*MYB28* is a transcription factor responsible for activating genes involved in aliphatic GSL biosynthesis. Downregulating *MYB28* might reduce the aliphatic GSL biosynthesis. RNAi-mediated knockdown of the *MYB28* decreased aliphatic GSL profiles, but the indolic GSLs remained unchanged (Gigolashvili et al., 2007). MYB28, MYB76, MYB29 activate genes in the aliphatic GSL biosynthetic pathways, such as *MAM3*, *CYP79F1*, and *CYP83A1*, without affecting genes involved in the indolic GSL pathway. Conversely, MYB34 and MYB51 activate genes for the indolic GSL biosynthesis (Gigolashvili et al., 2007). The downregulation of these transcription factors might affect genes such as *CYP79B2* and *CYP83B1* and prevent their conversion from tryptophan to GSLs. However, this might negatively affect the plant’s auxin homeostasis due to the interconnected nature of these pathways.

Moreover, the role of GSLs in defense must be addressed. Knocking down all transcription factors might harm the plants due to their interconnection with phytohormones, defense, growth and development. Thus, manipulating GSL profiles in specific tissues could be a better approach to maintaining the plant defense intact.

Biosynthesis and accumulation of GSLs show a distinct spatiotemporal pattern (Jorgensen et al., 2015) facilitated by glucosinolate transporters (GTR) transporting GSLs to various tissues. GTR proteins belong to the family of nitrate transporter 1/peptide transporter (NRT1/PTR). GTR1, GTR2, and GTR3 were recently identified, and elaborate information on GSLs mobilization and accumulation in sulfur-rich cells (S-cells) for storage in *A. thaliana* was described by (Schuster et al., 2006; Nour-Eldin et al., 2012). The manipulation of the GSL profiles via their transporters could be a better option. The *gtr1gtr2* double knockout mutant failed to transport GSLs to seeds, resulting in a tenfold accumulation of GSLs in source tissues such as leaves and silique walls. The absence of aliphatic and indole GSLs in the *gtr1gtr2* double knockout mutant seeds indicates their role in phloem loading and long-distance GSL transport. Thus, disrupting these transporters effectively reduces GSLs in the seeds without compromising plant defense.

**Figure 5:**
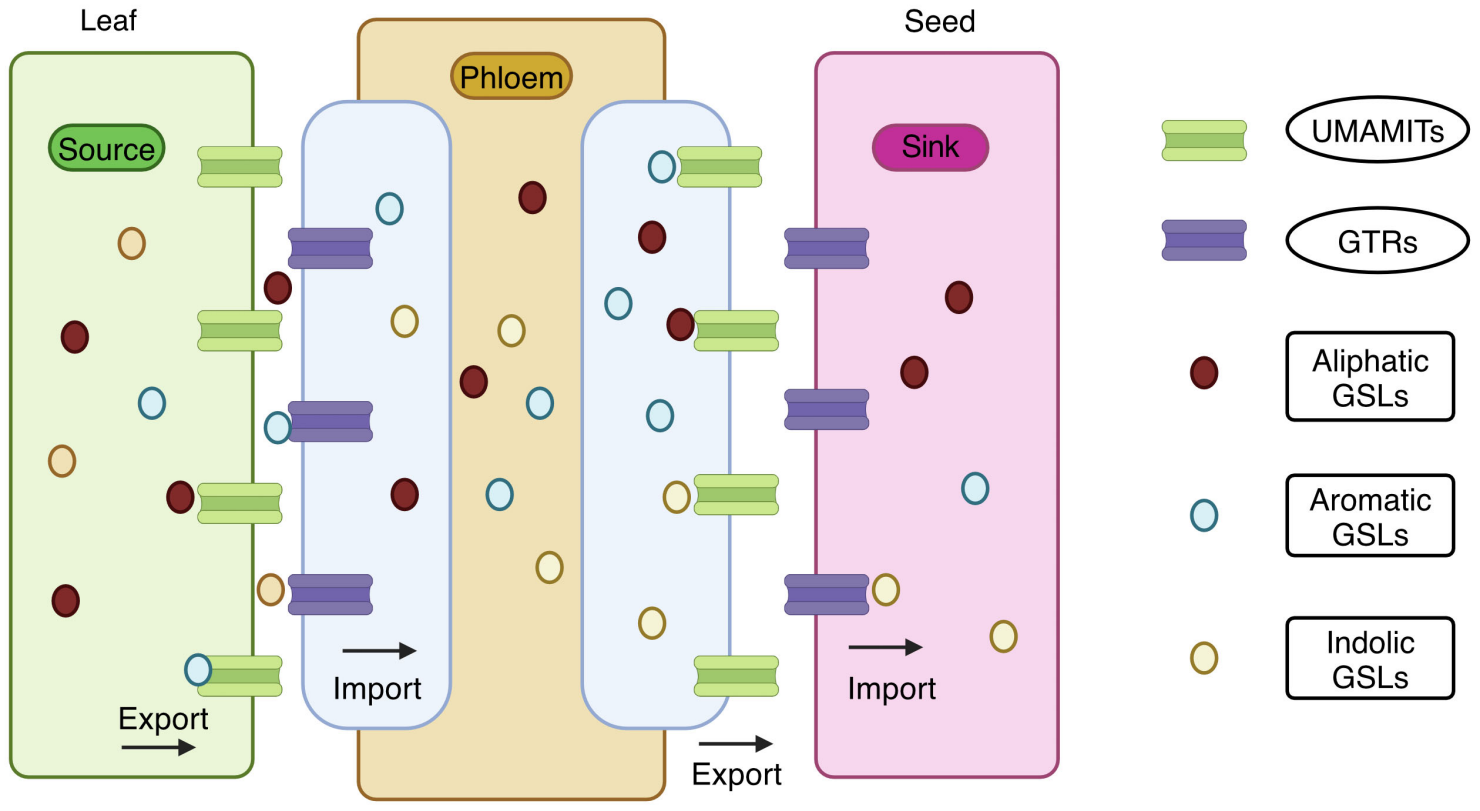
Transport of glucosinolates from source tissues to sink tissue. Usually Multiple Amino Acids Move in And Out Transporter (UMAMIT) acts as an exporter and mobilizes the glucosinolates from the source tissues into the apoplast. Glucosinolate transporters (GTR) acts as an importer and imports the glucosinolates into the phloem channel. Again, UMAMIT exports the glucosinolates into the apoplast at the unloading zone, and GTR imports them into the sink tissues. Both UMAMIT and GTR are involved in the transport of aliphatic, aromatic, and indolic glucosinolates in the plant (Created with BioRender.com).

This was successfully demonstrated in *Brassica juncea* (Mann et al., 2023). Knocking out *GTR1* and *GTR2* reduced the seed GSL content in *B. juncea* from 146.09 μmoles/g DW to 6.21 μmoles/g DW without compromising the defense (Mann et al., 2023). Similarly, the knockout in *Camelina sativa* reduced the seed GSL content up to 85%-88% (Hölzl et al., 2023). A clear picture of the GSL transporter is given in **Figure 5** (Xu et al., 2023; Sanden et al., 2024). They have reported that the GTRs function as importers, and UMAMIT (Usually Multiple Amino Acids Move in And Out Transporter) functions as an exporters; together, they function as GSL transporters.

UMAMIT are localized in the plasma membrane and export GSLs into the apoplast from the source tissue. In contrast, GTRs in phloem companion cells import GSLs into the phloem, facilitating the transport to sink tissues. At the phloem unloading zone, GSLs are exported by UMAMIT to the apoplast, and the GTRs import the GSLs into the seed. Both transporters transport all three types of GSLs: aliphatic, aromatic and indolic. Targeting UMAMIT genes impairs GSL export and prevents deposition in the sink tissues. UMAMIT29, UMAMIT30, and UMAMIT3 are exporters found in the *Arabidopsis thaliana* (Xu et al., 2023). The *umamit29 umamit30 umamit31* triple mutants exhibited low seed GSLs. In the seeds, mutant alleles of *UMAMIT29* showed an 80% reduction in total GSL levels. Thus, *UMAMIT29* could be the optimal target gene for reducing the GSL export.

#### 5.4.4 Phytic acid

Reducing phytic acid content in rapeseed is essential for improving the nutrient’s bioavailability. Phytic acid is synthesized through lipid-dependent and lipid-independent myo-inositol pathways. PMT5 and PIP5Ks play a vital role in the lipid-dependent biosynthesis pathway, whereas ITPK is pivotal in the lipid-independent biosynthesis pathway. SAC8 acts as a phosphatase and regulates phosphoinositide (Zhong and Ye, 2003). PMT5 (Polyol/monosaccharide transporter) is involved in transporting myo-inositol, glycerol, and several hexoses and pentoses, including ribose (Klepek et al., 2005). Thus, regulating the transporter could reduce the phytic acid content in the seeds. However, a major limitation lies in the multiple roles of the transporter. The knockdown mutants might result in poor seed development since PMT5 transports other compounds like glycerol and ribose. Thus, knockdown of the biosynthesis genes could be a better solution.

Phosphatidylinositol 4-phosphate 5-kinases (PIP5Ks) are a group of kinases that catalyze the production of phosphatidyl inositol (4,5)-bisphosphate (Van den Bout and Divecha, 2009). The *ITPK* gene family in *B. napus* holds four paralogues, namely *BnITPK1*, *BnITPK2*, *BnITPK3*, and *BnITPK4*. Ethyl methanesulphonate (EMS)-induced mutant lines targeting six genes in the phytic acid biosynthesis pathway were done by (Sashidhar et al., 2020a). However, due to the polyploid nature of rapeseed, the desired phenotype carrying all the mutant alleles requires laborious crossing. To overcome these challenges, *BnITPK1* and *BnITPK4* were knocked out by using CRISPR, resulting in a 35% phytate reduction (Sashidhar et al., 2020b). Similar phytate reduction has also been reported in soybean (Song et al., 2022a) and wheat (Ibrahim et al., 2022) using the CRISPR/Cas9 system.

#### 5.4.5 Sinapines, lignins, cellulose and hemicellulose

Sinapine and lignin biosynthesis are interconnected and produced through the phenylpropanoid pathway (Boerjan et al., 2003; Milkowski and Strack, 2010). This interconnection provides a strategic advantage to alter both profiles by editing specific genes involved in biosynthesis. The enzymes involved in sinapine biosynthesis are UDP glucose sinapate glucosyltransferase (SGT), sinapoyl glucose choline sinapoyl transferase (SCT), sinapoyl glucose L-malate sinapoyl transferase (SMT) and sinapoyl choline (sinapine) esterase (SCE) (Milkowski and Strack, 2010). RNAi-mediated silencing was done in *B. napus* for the *UGT84A9* gene, which disrupted the amounts and nature of the phenylpropanoid end products (Wolfram et al., 2010). Similarly, RNAi-mediated *BnREF1* suppression resulted in reduced levels of sinapate esters, conjugated monolignols, dilignols, and trilignols and altered the minor conjugates of ferulate, caffeate, and 5hydroxyferulate (Mittasch et al., 2013).

The polymerization of monolignols like p-coumaryl alcohol, coniferyl alcohol, and sinapyl alcohol is essential for lignin biosynthesis (Xie et al., 2018). The monolignols are synthesized in the cytoplasm and transported to the cell wall, where they are polymerized into lignin, creating the p-hydroxyphenyl (H), guaiacyl (G) and syringyl (S) units (Boerjan et al., 2003). Targeting genes involved in this biosynthetic pathway, such as *quinate hydroxycinnamoyl transferase (HCT), caffeic acid O-methyltransferase1* (*COMT1*), *ferulate-5-hydroxylase*, *BGLU46, BGLU45, cinnamyl alcohol dehydrogenase, phenyl ammonia lyase 4 and cinnamoyl-CoA reductase 1* could reduce the lignin contents. The mutant obtained through CRISPR/Cas9 editing for *caffeic acid O-methyltransferase 1* reduced lignin content by 14% and 34% increase in higher fermentable glucose recovery rate than the wild-type (Lee et al., 2021).

Additionally, cellulose synthase and mannose-1-phosphate guanylyltransferase 1 are involved in cellulose and hemicellulose biosynthesis, respectively (Lukowitz et al., 2001; Hu et al., 2018; Bhinder et al., 2022). This type of genetic modification improves the use of rapeseed in biofuel production by altering its fiber fraction, which comprises lignin, cellulose and hemicellulose. These compounds are involved in plant defense and maintain cell wall integrity to provide mechanical support to the plants. In this regard, promoter editing could be used to alter their levels rather than knocking down the genes completely, to maintain the essential structural integrity and defense while optimizing biosynthetic pathways.

#### 5.4.6 Seed storage proteins

Recent efforts have been focused on altering SSPs to improve the meal quality of rapeseed. Unlike napin, a cysteine-rich SSP in rapeseed, the SSP of Brazil nut is rich in methionine. A chimeric gene with a methionine-rich 2S SSP coding sequence with soybean lectin promoter was used to improve the methionine content in rapeseed (Guerche et al., 1990). A similar attempt was made using a phaseolin promoter, resulting in an increase of 33% in the methionine content (Altenbach et al., 1992).

Improving the transport of the amino acids to the seed (sink) is another possible strategy. In arabidopsis, CAT6, AAP1, AAP2 and AAP8 have been reported to transport the amino acids from the source to the sink. CAT6 preferentially is known to transport neutral and cationic amino acids (Hammes et al., 2006). The mutant *aap1* reduced total nitrogen (N) but showed a substantial increase in free amino acids in the seed coat and endosperm. This indicates that *AAP1* is involved in the import of amino acids into the embryo (Sanders et al., 2009). AAP2 is involved in the phloem loading of amino acids; the *aap2* mutant showed decreased amino acids in phloem sap and increased N content, leading to more branches (Zhang et al., 2010a). Meanwhile, AAP8 is crucial in transporting amino acids into the endosperm to nourish the embryo during early embryogenesis, with a high affinity towards acidic amino acids (Schmidt et al., 2007).

Altering a particular transporter could increase specific amino acid content. For instance, upregulating the expression of *AtCAT6* could improve the transport of cationic amino acids like lysine and neutral amino acids like methionine and cysteine. Thus, increasing the expression of transporters in seeds could enhance the levels of essential amino acids such as lysine and methionine, which are often limited in plant-based diets (Yang et al., 2023).

Silencing cruciferin genes in rapeseed increased SSP cysteine, lysine, and methionine levels (Kohno-Murase et al., 1995). Conversely, silencing the napins in rapeseed resulted in higher levels of cruciferins and lower amounts of cysteine and lysine (Kohno-Murase et al., 1994). The inhibition of one type of SSP results in the accumulation of free amino acids, which can be directly allocated to synthesizing another SSP (Kohno-Murase et al., 1995). This suggests that protein profiles can be altered by targeting the specific genes involved in the biosynthesis of SSPs. However, these alterations did not affect the seed’s overall protein or oil content (Rolletschek et al., 2020). Thus, targeting SSP biosynthesis genes could modify the proportion of cruciferin and napins and improve their implications in food processing industries.

## 6 Conclusion

The increasing need for alternative protein sources due to rising population growth demands the exploration of new and sustainable options. Plant-based diets are gaining popularity, highlighting the need for alternative plant-derived proteins. Rapeseed byproducts hold promising potential as an alternative protein candidate for human food and animal feed. Their balanced amino acid profiles and functional properties make them suitable for various applications. However, the presence of ANFs poses challenges to their utilization. Strategies to reduce ANFs, including genetic, biotechnological and microbial methodologies, are essential to enhance rapeseed byproducts’ nutritional quality and acceptability. Therefore, further research and development are needed to optimize these strategies and fully utilize the potential of rapeseed as a sustainable protein source.
